# Assessment of airborne pollutants in wastewater treatment plants

**DOI:** 10.1007/s11356-025-36484-3

**Published:** 2025-05-07

**Authors:** Gabriela Viteri, Alfonso Aranda, Yolanda Díaz-de-Mera, Ana Rodríguez, Nuria Rodriguez-Fariñas, Diana Rodríguez

**Affiliations:** 1 Facultad de Ciencias y Tecnologías Químicas, Departamento de Química Física, Universidad de Castilla-La Mancha, Avda. Camilo José Cela 1B, 13071 Ciudad Real, Spain; 2 Facultad de Ciencias Ambientales y Bioquímica, Departamento de Química Física, Universidad de Castilla-La Mancha, Avenida Carlos III s/n, 45071 Toledo, Spain

**Keywords:** Wastewater treatment plant, Criteria air pollutants, Particles, Trace elements, Volatile organic compounds, Environmental indicators and health risk

## Abstract

Wastewater generation rates have increased considerably in recent years due to population growth, urbanisation, and industrialisation. Although wastewater treatment plants (WWTPs) play a crucial role in purifying wastewater and mitigating industrial and domestic pollution in global freshwater reserves, their impact on air quality in nearby areas is frequently neglected. Therefore, this study assessed air quality in two WWTPs in Toledo, Spain. One is located in an industrial area and processes both industrial and domestic sewage (WWTP1), and the other is situated in a rural area and treats domestic wastewater (WWTP2), handling approximately double the treatment flow. Moreover, the aeration systems in the bioreactors differ. Measurements were made over a period of 6 months and included levels of gaseous emissions, such as SO_2_, CO, NOx, O_3_, volatile organic compounds (VOCs), particulate matter (PM_2.5_ and PM_10_), and trace elements (TEs). Environmental and health risk indicators were calculated to estimate the impact of exposure to air pollution on nearby ecosystems and the population’s health, respectively. The study revealed a low contribution of the WWTPs to the SO_2_, CO, and PM levels. However, WWTP1 showed an NOx annual average concentration close to the legislated threshold, and the target O_3_ value for the protection of human health was also exceeded. Regarding VOCs, the main contribution was the group of oxygenated aromatics. Concerning TEs, a high enrichment factor value was obtained for sodium, arsenic, selenium, potassium, nickel, copper, zinc, and lead at WWTP1 and for zinc, cadmium, copper, and selenium at WWTP2.

**Supplementary Information:**

The online version contains supplementary material available at 10.1007/s11356-025-36484-3.

## Introduction

Water is a fundamental natural resource that is essential for life, livelihood, food security, and sustainable economic development (Somlyòdy and Varis 2006). According to the 2018 edition of the United Nations World Water Development Report, nearly 6 billion people will suffer from clean water scarcity by 2050 (WWDR 2018). This is the result of different anthropogenic activities linked to climatic change and the growing population size. With the rapid expansion of the population, cities, and industries, a large volume of wastewater is expected to be generated. The average daily volume of wastewater generated by human activities depends on water availability, cultural factors, the cost of the water, and the economic conditions (Kalavrouziotis and Kalavrouziotis 2015). Wastewater treatment, also called sewage treatment, is a fundamental part of global urbanisation as it removes impurities and pollutants through a series of biological and physical processes in distinct reactors, producing water of an acceptable quality, which is released back into the environment. Thus, the establishment of wastewater treatment plants (WWTPs) significantly reduces untreated wastewater released into the environment through several stages. These multi-stage processes release different categories of pollutants to the atmosphere (Al-Dosary et al. 2015; Mamais et al. 2015). WWTPs are a source of greenhouse gases (GHGs), such as methane (CH_4_), nitrous oxide (N_2_O), and carbon dioxide (CO_2_) (Wang et al. 2022; Zhou et al. 2022), volatile organic compounds (VOCs), metals, and particulate matter (PM) (Du et al. 2020; Upadhyay et al. 2013). Several studies have focused on the characterisation and quantification of GHGs (Zhou et al. 2022; Stadler et al. 2021; Caivano et al. 2017; Foley et al. 2015). However, the emission of criteria pollutants, VOCs, PM, and metals has been less extensively measured. For instance, during ammonium nitrification by ammonium-oxidising bacteria, NO_2_ is produced, and NO is formed by the oxidation of hydroxylamine to NO_2_. Moreover, the nitrite denitrifier activity with hydrogen, ammonium, or pyruvate as an electron donor produces NO (Foley et al. 2015; Gustavsson and Cour Jansen 2011). Moreover, VOCs can also be released into the atmosphere through volatilisation during aeration treatment (Yang et al. 2014), while PM can be emitted by aeration processes (Upadhyay et al. 2013) or due to mechanical action (Liu et al. 2020; Yang et al. 2019). Furthermore, the residual matter from the treatment processes, i.e. sewage sludge (SS), could contain harmful substances, such as heavy metals, and poorly biodegradable organic compounds (Seggiani et al. 2012). Consequently, pollutants released from wastewater treatment or by-products, such as sludge, could contribute to air quality deterioration. Thus, this study aimed to identify criteria pollutants, PM, trace elements (TEs), and a broad range of VOCs to explain the impacts of WWTPs on air quality.

## Materials and methods

### WWTP description and sample collection

The air quality of two municipal WWTPs in Toledo, Spain, was evaluated based on certain atmospheric pollutants. Toledo is a city situated on a rugged promontory surrounded on three sides by the Tagus River, 73 km southwest of the Spanish capital, Madrid. It covers an area of 232.1 km^2^ and has 86,070 inhabitants (INE 2023). Toledo has a continental Mediterranean climate, with a low level of rainfall concentrated in spring and at the end of autumn (Kottek et al. 2006).

From August 2021 to January 2022, air samples were obtained from a WWTP located in a residential and industrial neighbourhood of Toledo (denoted as WWTP1). WWTP1 has a capacity of 24,000 m^3^ sewage/day, although the average capacity currently is 8000 m^3^/day. The plant receives domestic (60%) and industrial (40%) sewage. From February 2023 to July 2023, air samples were collected in another WWTP (denoted as WWTP2), which is in a rural area of Toledo. WWTP2 has a capacity of 36,000 m^3^ sewage/day, although the real average capacity currently is 13,000 m^3^/day. The plant receives only domestic sewage from the remaining neighbourhoods of Toledo. A description of WWTP1 and WWTP2 and the location of measurement points is shown in Fig. S1. Both WWTPs also differ in the aeration treatment in the bioreactor. At WWTP1, the oxygen supply is made by means of mechanical agitation on the surface, which moves the whole water mass. However, WWTP2 used a series of submerged grills (membrane diffusers) that cover the entire bottom surface of the aeration tank, making agitation more distributed and controlled (Rizzardi et al. 2023).

The results of both WWTPs were compared to the values monitored by the air quality station of Toledo. This suburban background air quality station is in a residential and commercial area, and consequently, the main emission source that affects this station is traffic and combustion. The station is around 6.1 km away from WWTP1 and 8.8 km away from WWTP2 (Fig. S1) and serves as a reference (JCCM 2020a, 2020b).

### Instrumentation

Our previous studies have provided detailed descriptions of the air quality monitoring equipment used in the present study (Viteri et al. 2023, 2024), and only a brief description is given here. The equipment used is based on the official methods included in Annex VII of R.D. 102/2011, of 28 January, relative to air quality improvement.

Prior to starting each campaign in both WWTPs, the analysers were calibrated to ensure optimal data quality. Calibration was carried out by Envira, which has ENAC accreditation to act as an accredited laboratory according to UNE-EN ISO/IEC 17025:2005. Moreover, maintenance, such as changes in filters and verification, was carried out every month.

#### Gases (criteria pollutants)

O_3_ was measured using a UV-absorption technique (T400, Teledyne), and CO was determined using the reference method based on IR absorption (T300, Teledyne). SO_2_ was monitored with an instrument based on the standard UV fluorescence technique (T100U, Teledyne), and NOx (NO + NO_2_) was measured by chemiluminescence detection (T200P, Teledyne). All the equipment was installed inside a thermally regulated cabin with a sampling manifold to maintain a constant working temperature of 22 ± 2 °C. Continuous data were used to build 10-min and hourly average data files.

VOCs were collected on sorbent stainless steel multi-bed tubes filled with Tenax TA/Carbograph 1 TD/Carboxen 1003 (Markes MKC3 AAXX5266 for analytes in the range C_2/3_ to C_30/32_). An MTS-32 multi-tube sequential sampler (Markes) was used for these collections, with active sampling performed according to the reference EPA TO-17 method (EPA TO-17 1999). Each campaign consisted of the collection of 28 samples (4 per day in each week) with pumping air volumes of approximately 25 mL min^−1^. Additionally, four tubes were also placed in the system throughout the whole campaign without flow to serve as references. The tubes were thermally desorbed using a Shimadzu TD-30R thermal desorption system and analysed with a Shimadzu GC-MS TQ8040 triple quadrupole mass spectrometer for the identification and quantification of analytes in the air samples. A DB-624UI GC column (60 m × 0.25 mm × 1.4 mm) was employed for the analysis. For each campaign week, a calibration curve was obtained by preparing a set of eight diluted samples from a commercial calibration mix (8260 Restek) containing the certified concentration (2000 µg mL^−1^) of 76 pollutants. The analysis procedure and the average detection limit for each component have been previously reported (Viteri et al. 2023). The detection limit for the studied compounds was in the range of 2×10^−3^–5×10^−2^ µg m^−3^.

#### PM_2.5_ and trace elements

The mass concentrations of PM_2.5_ and PM_10_ were measured using the β-ray absorption method (BAM 1022 instrument, Met One). Moreover, PM_2.5_ was sampled on glass fibre filters (Whatman, 47 mm) using a low-volume Comde-Derenda sampler device (2.3 m^3^ h^−1^) for TE analysis. TEs suspended on PM_2.5_ were determined according to standard UNE-EN-14902: 2006. The concentrations of sodium (Na), aluminium (Al), potassium (K), calcium (Ca), iron (Fe), zinc (Zn), nickel (Ni), arsenic (As), cadmium (Cd), lead (Pb), chromium (Cr), cobalt (Co), manganese (Mn), copper (Cu), magnesium (Mg), mercury (Hg), and selenium (Se) were analysed using a triple quadrupole ICP-MS iCap-TQ instrument (Thermo Electron Corporation, Germany). The conditions are summarised in Supplementary Data, Text S1, and Table S1. The detection limits and metal concentrations in the blank filter sample are detailed in Supplementary Table S2.

### Air mass back trajectory analysis

The analysis of the origin and frequency of air masses was simulated using the online National Oceanic and Atmospheric Administration HYbrid Single Particle Lagrangian Integrated Trajectory (HYSPLIT) software (Stein et al. 2015). In this study, two types of back trajectories were calculated: (a) for PM_2.5_-bound TEs based on a 7-day sampling period at a height of 1000 m (Bouchlaghem et al. 2021), (b) for dust intrusions over 2 days at altitudes from 1000 to 3000 m (Escudero et al. 2005, 2016). These trajectories were launched at 14 UTC every 12 h, and a maximum of 14 trajectories were generated to capture variability during the measurement period. These ensembles of trajectories were designed to represent the atmospheric boundary layer conditions from which the air masses originated. The Lagrangian trajectories were driven by GFS meteorological fields with a resolution of 0.25° (Kwok et al. 2017).

### Environmental and health risk indicators

The ozone formation potential (OFP), the secondary organic aerosol formation potential (SOAFP), the carcinogenic risk (CR) and non-carcinogenic risk in terms of the hazard index (HI), the enrichment factor (EF), and the potential ecological risk index (RI) were calculated to assess the potential hazards posed by the studied pollutants to both ecosystems and human health. More information about the environmental and health risk indicators is included in the Supplementary Data (Text S2).

### Statistical analysis

Statistical analyses were conducted using SPSS (IBM SPSS Statistics 24). Before performing statistical analyses, the normality of the data distribution was assessed using the Kolmogorov–Smirnov test. Parametric tests (analysis of variance (ANOVA) and Student’s *t* test) or non-parametric tests (Mann–Whitney *U* and Kruskal–Wallis tests) were performed depending on data normality. Spearman’s correlation test was used to determine the relationships between the parameters. For all statistical tests, a significance level of *p* < 0.05 was used, while correlation tests showed a lower significance threshold at *p* < 0.01.

## Results and discussion

### Sulphur dioxide and carbon monoxide

The SO_2_ concentrations at WWTP1 ranged from 1.4 to 2.2 μg m^−3^, with a mean value of 1.8 ± 0.1 μg m^−3^. In contrast, at WWTP2, the concentration ranged from 7.8 to 12.1 μg m^−3^, with a mean value of 9.5 ± 0.1 μg m^−3^. At both WWTPs, the lowest values were recorded during the cold season (December and January in WWTP1 and February in WWTP2), while the highest values were achieved in summer (August in WWTP1 and July in WWTP2). This seasonal variation was also registered in the air quality station, with statistically significant differences between the warm and cold seasons (*p*-value < 0.05). Emission sources, such as traffic and/or industrial activities, could be the cause of this behaviour. In an Iraqi WWTP, Al Kindi et al. (2022) similarly found the highest mean concentration during the summer season near an aeration tank or bioreactor. SS contains sulphur in a range of 0.3–2.3 wt% (Dewil et al. 2008). During SS thickening, the oxygen level decreases due to microbial activity, leading to the gradual reduction of sulphates to sulphides. As a result, in sludge dewatering, there is a minor fraction of sulphur in the sludge water because sulphates have already been reduced to insoluble sulphides, mainly hydrogen sulphide (H_2_S) (Dewil et al. 2008, 2009). H_2_S is one of the main precursors of SO_2_ in the atmosphere (Wayne 2000); thus, it has been determined that the H_2_S concentrations emitted from water highly contaminated with organic sulphur compounds are correlated with the ambient SO_2_ concentrations (Muezzinoglu 2003).

**Fig. 1:**
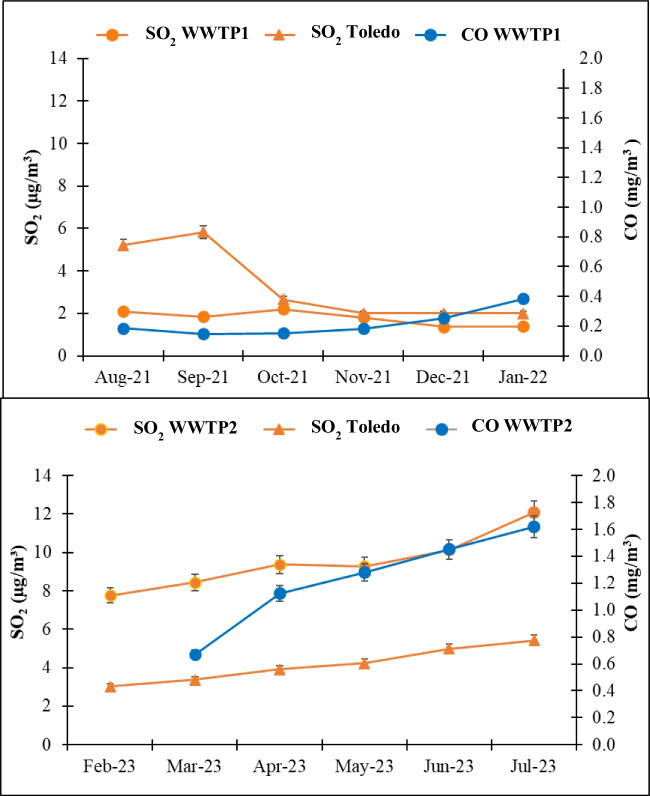
Monthly average SO_2_ and CO (right axis) concentrations at the wastewater treatment plants (WWTPs) and the air quality station of Toledo.

The levels recorded at the Toledo air quality station were significantly higher than those measured at WWTP1 but lower than those measured at WWTP2 (*p*-value < 0.05) (Fig. 1). The higher SO₂ concentrations measured at WWTP2 could be due to its significantly greater wastewater flow, which is approximately double that of WWTP1. Moreover, WWTP1 utilises deodorisation towers with a NaOCl/NaOH solution for chemical air washing, effectively reducing the emission of sulphur-containing compounds, while WWTP2 does not employ this method. The daily limit of 125 µg m^−3^ established by Spanish statutory thresholds R.D. 102/2011 (dated January 28, in relation to air quality) has never been exceeded.

The weekend effect of SO_2_ concentrations was analysed in both WWTPs to detect the influence of human-related activities. SO_2_ concentrations were significantly higher on weekdays only at WWTP1 (*p*-value < 0.05, Fig. S2). This could be due to the contribution to SO_2_ emissions from traffic and/or industrial activities close to WWTP1.

However, the CO concentrations measured at WWTP1 ranged from 0.15 (September and October) to 0.38 mg m^−3^ (January), with a mean value of 0.22 ± 0.02 mg m^−3^. In contrast, at WWTP2, the concentration ranged from 0.67 (March) to 1.62 mg m^−3^ (July), with a mean value of 1.20 ± 0.02 μg m^−3^ (Fig. 1). No data were available in February at WWTP2 due to a fault in the analyser. The values at WWTP1 ​were quite consistent each month, but they were somewhat higher in December and January, probably due to vehicles and heating systems. However, at WWTP2, values increased from March to July, with maximum values achieved in July. At WWTP2, CO could be generated during aerobic digestion of organic waste, such as CO_2_ (Stegenta-Dąbrowska et al. 2019). Previous studies in Taiwan registered mean values of 0.75 ± 0.44 (Widiana et al. 2017) and 0.64 ± 0.40 mg m^−3^ (Widiana et al. 2019). These values are comparable to the concentrations measured at WWTP2. The Toledo air quality monitoring station does not measure CO; therefore, a comparison could not be made. The maximum daily 8-h mean value for CO (10 mg/m^3^) was not exceeded (R.D. 102/2011). The measurements recorded were 0.29 mg m^−3^ at WWTP1 and 0.18 mg m^−3^ at WWTP2.

A weekend effect was again observed at WWTP1 when plotting the average CO concentration for working days and Sundays for the different weeks of the study (Fig. S3). Statistical significance was observed (*p*-value < 0.05). These results suggest that CO levels may be influenced by an additional source, with traffic and industry being the most likely contributors.

### Nitrogen oxides

**Fig. 2:**
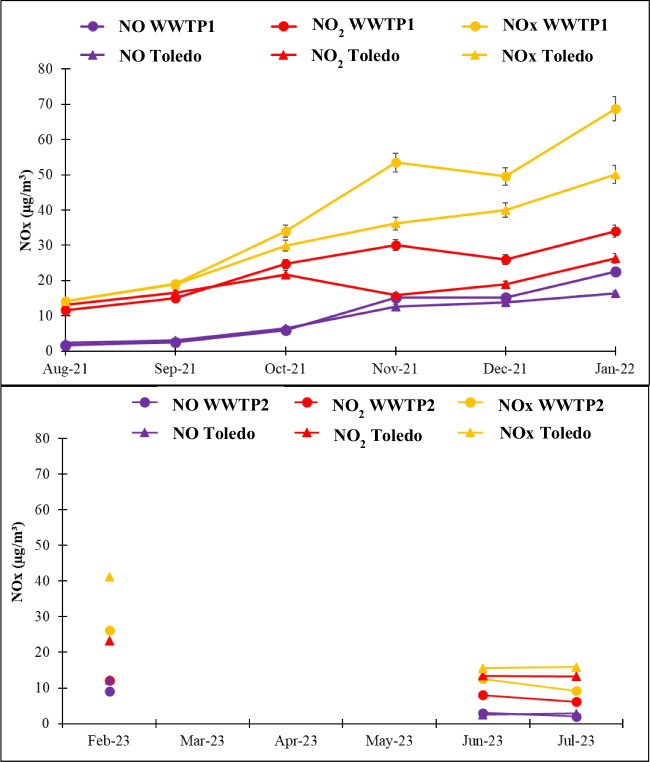
Monthly average NOx concentrations at the wastewater treatment plants (WWTPs) and the Toledo station

No data were available for March, April, or May at WWTP2 due to a fault in the analyser. At WWTP1, the monthly average NO and NO₂ concentrations ranged from 1.6 to 22.6 µg m^−3^ and from 11.7 to 34.0 µg m^−3^, respectively; the minimum and maximum values recorded in August and January, respectively. However, at WWTP2, NO and NO₂ concentrations ranged from 2.0 to 9.1 µg m^−3^ and from 6.1 and 12.2 µg m^−3^, with minimum and maximum values recorded in July and February, respectively. As shown in Fig. 2, NOx concentrations exhibited an increasing trend from the warm to the cold season for both WWTPs, with statistically significant seasonal differences (*p*-value < 0.05). These high concentrations observed in autumn and winter are often attributed to unfavourable meteorological conditions (Cai et al. 2017; He et al. 2017; Zhao et al. 2016), such as low temperatures, high relative humidity, a lower planetary boundary layer, stagnant wind conditions, and increased emissions from heating systems (Lei et al. 2022).

Thus, the average NOx concentration measured was 39.8 ± 0.4 µg m^−3^ at WTTP1 and 16 ± 0.4 µg m^−3^ at WTPP2. The average NO_x_ value achieved at WWTP1 was close to the annual limit value established by RD 102/2011 (40 µg m^−3^ expressed as NO_2_). NOx can be released during nitrogen removal in WWTPs (Fuerhacker et al. 2001). Nitrogen in wastewater is typically removed through biological treatment processes, specifically nitrification and denitrification. During ammonium nitrification, NO is produced in the bioreactor by ammonium-oxidising bacteria (Lipschultz et al. 1981). When NO is released into the atmosphere, it reacts with O₂ to form NO_2_. The NOx levels at WWTP1 were significantly higher than those measured at the monitoring station, while the values at WWTP2 were lower. WWTP1 is located in an urban area with traffic and industry, which can contribute to increased NOx emissions from the plant. Additionally, colder temperatures tend to increase fossil fuel combustion for heating and often lead to greater use of personal vehicles for transportation. In contrast, WWTP2, located far from residential and industrial areas, showed the lowest NOx values, likely resulting from biological treatment processes and sludge management.

In the case of NOx, weekend effects were observed at both WWTPs (Fig. S4), showing statistically significant differences (*p*-value < 0.05). However, this effect is more noticeable at WWTP1, probably due to the traffic and industry close to this plant.

Regarding the hourly behaviour of NOx, two maxima were observed for the overall months (especially at WWTP1), one in the early morning, and the other in the late afternoon (Fig. S5). Both NOx maxima coincided with peak-hour traffic, mainly at WWTP1 due to its location, and at WWTP2 between entry and exit of employees. Moreover, these peaks coincided with an increase in flow wastewater rates, which reached WWTPs due to residential areas.

### Ozone

**Fig. 3:**
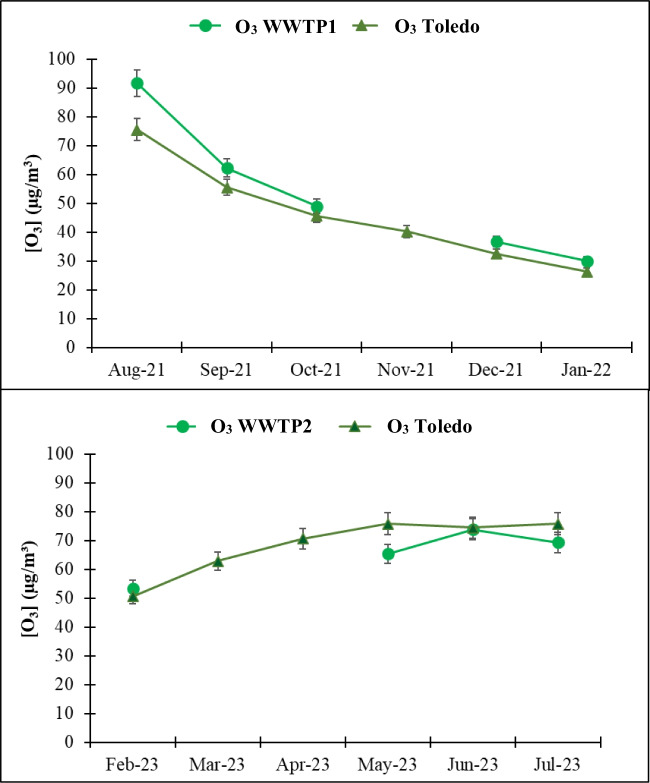
Monthly average O_3_ concentrations at the wastewater treatment plants (WWTPs) and the Toledo station.

No data were available for November at WWTP1 and for March and April at WWTP2 due to faults in the analyser. During the months with data available, the monthly average O_3_ concentrations at WWT1 ranged between 30.1 and 91.7 µg m^−3^, with an average value of 68.1 ± 0.6 µg m^−3^. In contrast, at WWTP2, the concentrations ranged from 53.5 to 73.8 µg m^−3^, with an average value of 65.5 ± 0.6 µg m^−3^. Fig. 3 shows the monthly O_3_ concentrations of the Toledo air quality station and WWTPs. The objective value for human protection, set at 120 μg m^−3^, was reached on 27 days at WWTP1 and 9 days at WWTP2 over 6 months of measurement. However, this level should not be exceeded for more than 25 days in any calendar year (R.D. 102/2011). Therefore, there is a potential risk to both the population and the personnel working at these facilities.

At both WWTPs, O_3_ concentrations were higher during the warm season and decreased in the cold season (*p*-value < 0.05), exhibiting a pattern similar to that observed at the Toledo air quality station. Both WWTPs showed significant statistical differences with the Toledo station for O_3_ (*p*-value < 0.05). In contrast to NOx, O_3_ levels reached their maximum concentrations in spring and summer. In autumn and winter, when the solar irradiation intensity decreased, the O_3_ concentration in the surface atmosphere decreased. Fig. S6 shows the hourly profiles of O_3_ for two characteristic months of summer and winter, with a peak between 14 and 15 UTC when solar radiation is more intense, and temperatures are higher. Moreover, ground level O_3_ concentrations depend on the absolute and relative concentrations of its precursors and the intensity of solar radiation (Abdul-Wahab and Bouhamra 2004). O_3_ formation increases with increasing VOC concentrations, while rising NOx levels increase or decrease O_3_ depending on the prevailing ratio between VOCs and NOx (Seinfeld and Pandis 2006). The slightly higher O₃ values found at WWTP1 could be attributed to the UV disinfection process in the tertiary stage. Here, water is further purified by removing any remaining pollutants and impurities to meet specific quality standards before it is discharged back into the environment or reused. Both WWTPs use UV disinfection, i.e. water is exposed to UV light, which damages the genetic material of microorganisms, making them unable to replicate and causing their destruction (González et al. 2023).

At WWTP1, this process is used most frequently, particularly during the warm season, for gardening purposes rather than solely for the plant’s own use, as is the case with WWTP2. UV irradiation can also facilitate in situ O₃ formation, as UV light can generate ozone from atmospheric oxygen at wavelengths shorter than 240 nm (Alahdal et al. 2021).

### VOCs

A week-long campaign was carried out each month to sample VOCs at WWTPs: September and December at WWTP1 and February, March, April, and June at WWTP2. Samples were collected every 6 h at four different time intervals (Table S3). The tandem MS analysis enabled the identification and quantification of 51 VOCs at WWTP1 (with concentrations between 0.002 and 25 μg m^−3^) and 60 VOCs at WWTP2 (with concentrations between 0.004 and 17.8 μg m^−3^) out of 76 target pollutants. The remaining compounds in the reference standard were below the detection limits of the analytical method.

Regarding the hourly variation of VOCs, many of the compounds exhibited significant differences during the period from 12:00 to 18:00 (*p*-value < 0.05) at both WWTPs. Low concentration levels in the afternoon may be attributed to photochemical reactions that occur during this time, which are favoured by greater solar radiation and high temperatures. These conditions promote a reaction that eliminates VOCs to form ozone (Ho et al. 2004).

**Fig. 4:**
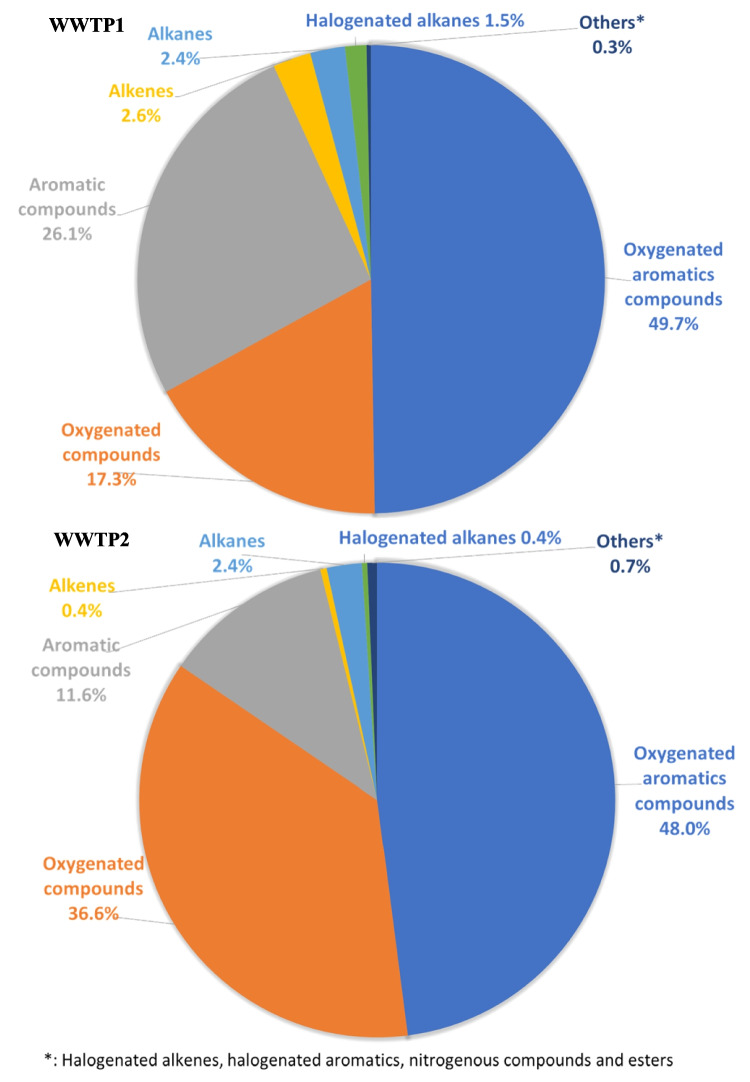
Average percentage contribution by type of volatile organic compound (VOC) release at both wastewater treatment plants (WWTPs)

As shown in Fig. 4, the largest contribution of VOCs identified in the WWTPs was from oxygenated aromatics group, accounting for 49.7% at WWTP1 and 48% at WWTP2. This was followed by oxygenated compounds, which made up 17.3% at WWTP1 and 36.6% at WWTP2, and aromatic compounds, which comprised 26.1% at WWTP1 and 11.6% at WWTP2. Most of these compounds likely originated from organic matter decomposition in wastewater, with significant emissions occurring in the aeration tanks (Hamoda 2006; Yang et al. 2014). However, emissions from traffic, especially at WWTP1, could not be ruled out. The presence of several VOCs detected in this work is consistent with previous studies carried out in other WWTPs.

At a WWTP in Taiwan, Widiana et al. (2017) found that the most abundant VOCs were toluene, ethanol, and acetone, with concentrations greater than 20 μg m^−3^. Other identified compounds included hexane, ethylbenzene, xylenes, and ethyl acetate, with aromatic compounds being the most prevalent. Similarly, a study conducted in Turkey by Dincer and Muezzinoglu (2008) reported that monoaromatics comprised 69% of the total VOCs, followed by sulphur compounds (14%), halogenated compounds (10%), and aldehydes (6%). A WWTP in Mexico (Ramírez et al. 2011) measured higher concentrations of styrene (48–574 μg m^−3^), chloroform (2.22–155 μg m^−3^), ethylbenzene (20.5–96.9 μg m^−3^), m-p xylene (35.3–136 μg m^−3^), and 1,2,3 trimethylbenzene (56.9–104 μg m^−3^) likely due to the facility processing industrial wastewater.

When analysing the weekend effect (Fig. S7), no significant changes were observed at either WWTP (*p*-value < 0.05), indicating that for these compounds, traffic and/or industrial emissions at WWTP1 were less relevant than for NOx, SO_2_, and CO.

VOCs can have a significant impact on human health and the environment; thus, their identification in the WWTPs can determine the environmental impact of both the individual and combined effects. Thus, Table S4 shows the calculated OFP, SOAPF, OIV, CR, and HI at both facilities.

The odour emitted by WWTPs is a social problem. Thus, OIV was obtained by dividing the average concentration of each VOC quantified by its odour threshold. Odour threshold values for VOCs were taken from Nagata (2003). As shown in Table S4, the major odour contributors identified were α-pinene and aromatics, highlighting toluene at WWTP1 (Schauberger et al. 2011). Moreover, toluene had the highest OFP value at both WWTPs, with an average value of 75.6 at WWTP1 and 12.8 at WWTP2. M-xylene, o-p xylenes, ethylbenzene, and 1,2,4-trimethylbenzene were also VOCs with high OFP values. Although ethylbenzene and 1,2,4-trimethylbenzene presented lower average concentrations at both WWTPs, they had a high MIR value. Thus, their OFPs were higher than the other compounds. Moreover, VOCs play a crucial role in SOA formation of SOA; thus, based on the SOAPF calculations (Eq. S3), toluene, benzene, ethylbenzene, and xylenes were the principal SOA contributors in the WWTP campaigns (Table S4).

Of the evaluated VOCs, 13 were considered carcinogenic groups by the International Agency for Research on Cancer (IARC). Benzene is in group 1 (carcinogenic to humans); styrene in group 2 A (probably carcinogenic to humans); carbon tetrachloride, ethylbenzene, chloroform, naphthalene, and 2-nitropropane in group 2B (presumably carcinogenic to people); and toluene, bromoform, and 1,1,2-trichloroethane in group 3 (there is no evidence that they cause cancer in humans). Equation 4 showed an insignificant CR for all VOCs (CR < 10^−6^), except benzene at WWTP2, with a value of 1.1510^−5^ (Table S4). Regarding non-cancer risk health effects, such as sensory irritation of the eyes, nose, and throat, none of the VOCs evaluated represented a potential health risk since the HI was less than 1 (Eq. S6) (EPA 2009; Ramírez et al. 2012).

In the air quality normative, only benzene has set a limit for the protection of human health. The annual mean value should not exceed 5 µg m^−3^ (R.D.102/2011). The average concentrations were 3.4 ± 1.7 μg m^−3^ at WWTP2, and 0.8 ± 0.2 μg m^−3^ at WWTP1. These values are below the legislated limit.

### PM

**Fig. 5:**
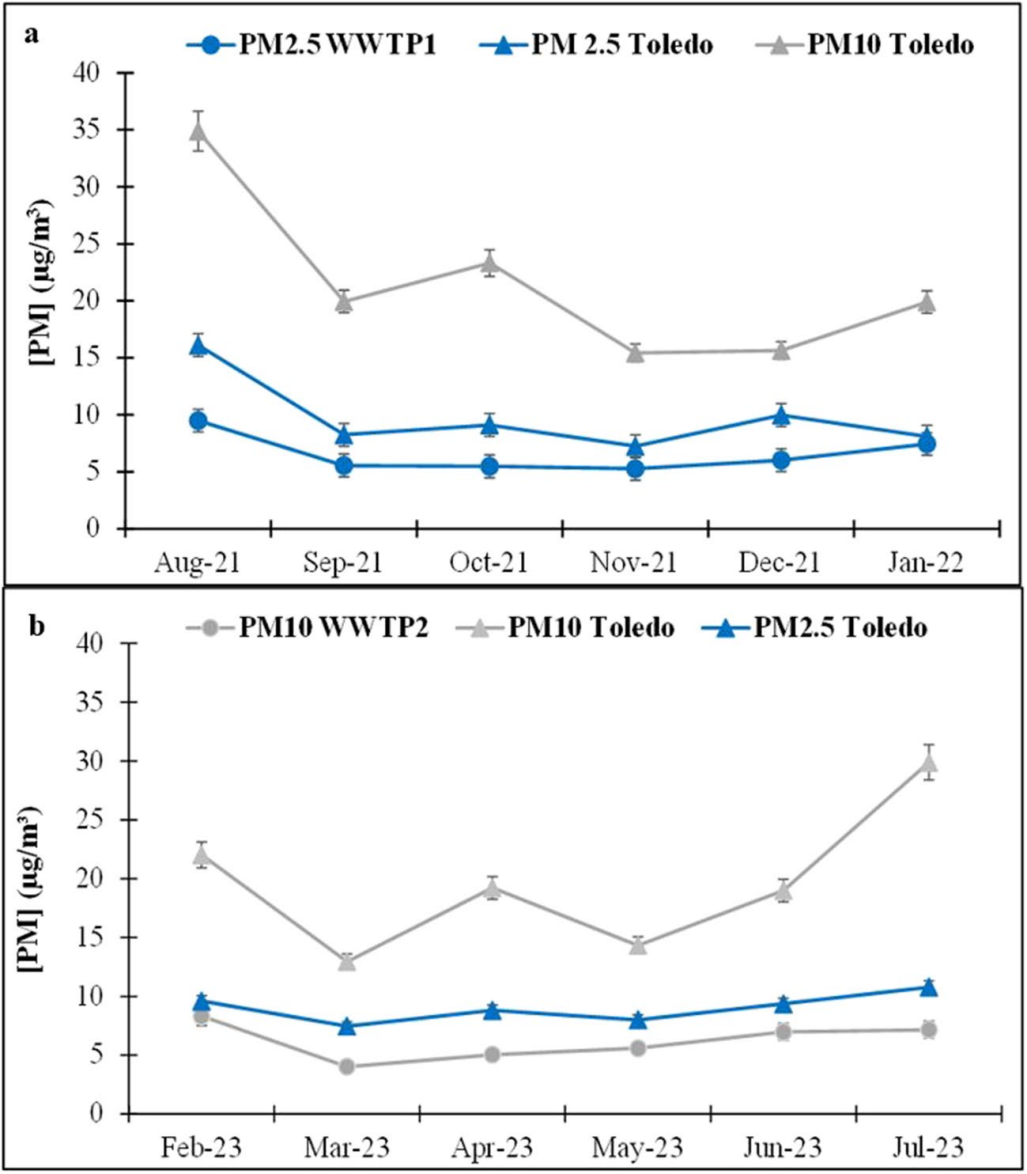
Monthly average particulate matter (PM) data at wastewater treatment plants (WWTPs) and air quality stations

The PM_2*.*5_ measurements at WWTP1 gave average values of 5.3 μg m^−3^ in November and 9.5 μg m^−3^ in August, with a mean value of 6.5 ± 1.0 μg m^−3^. These values were below the annual limit established by R.D. 102/2011 (20 μg m^−3^). Statistically significant differences were observed between the warm and cold seasons (*p*-value < 0.05) with larger values in the warm season (Fig. 5a). The higher number of days with Saharan dust intrusions observed during August 2021 (16 days) compared to the rest of the study months (MITECO 2022) may be the cause of the higher PM_2.5_ concentration measured in this season. This was confirmed with the back trajectory representations (Fig. S8), in which air masses from Africa were clearly observed during August 2021. At WWTP2, PM_10_ ranged from 4.0 μg m^−3^ in March to 8.3 μg m^−3^ in February, with a mean value of 6.2 ± 1.0 μg m^−3^ (Fig. 5b). These PM_10_ values ​were also lower than the annual limit established by RD102/2011 (40 μg m^−3^). No statistically significant differences were observed between the warm and cold seasons (*p*-value ˃ 0.05), despite that an increase in the relative humidity can increase the particle concentration. Thus, the PM concentrations were slightly higher in wetter months, such as December 2021 (RH = 78%) and January 2022 (RH = 74%) at WWTP1 and February 2023 (RH = 66%) at WWTP2, than in autumn (average RH = 65%) and spring (RH = 54%) at both WWTP1 and WWTP2. However, other factors, such as the suspension of PM of mineral origin in dry soil conditions and the intrusion of Saharan dust, led to higher concentrations in the warm season, counteracting the effects of humidity.

As shown in Fig. 5, although PM data for the studied period exhibited a behaviour similar to the Toledo air quality station, the concentrations were lower. Upadhyay et al. (2013) showed that the overall emissions of fine and coarse particles at WWTPs were small compared to other urban sources because they were removed by odour treatment units.

Since our sampling point was about 100 m from the aeration tanks (Fig. S1), we carried out punctual measures close to the bioreactor and at the sample point with portable PM equipment with different diameters (Aeroqual Series 200 model for PM_10_ and PM_2.5_, and model PCE-PQC 12EU for particles smaller than 1 μm). Average PM_1_, PM_2.5_, and PM_10_ concentrations measured in the aeration tanks at WWTP1 were four times higher than those measured at the usual sample point (Table S5). Thus, the diffuse aeration process that took place in WWTP1 resulted in the bursting of bubbles on the surface of the liquid, transferring particles and bioaerosols to the atmosphere (Piqueras 2017). However, in the case of WWTP2, there was no difference. These results are similar to those observed by Upadhyay et al. (2013), who showed that aeration tanks were an important source of particle emissions into the atmosphere and that the oxygen supply by means of membrane diffusers at WWTP2 is more distributed and controlled and emits less PM to the atmosphere.

Regarding the weekend effect, significant statistical differences (*p*-value > 0.05) were observed at both WWTPs, probably due to the decrease in traffic and industry at WWTP1 and a lower wastewater flow rate at both WWTPs (Fig. S9).

African dust intrusions were registered in the Iberian Peninsula (MITECO 2021, 2022, 2023) during the study period, indicating that these events could significantly contribute to the PM fraction at both WWTPs. By calculating back trajectory frequencies for the 48-h sampling period on days with high PM levels, we identified these events (Fig. S8), particularly at WWTP1, where Saharan intrusions significantly contributed to background particles.

### Trace elements associated with PM_2.5_

In addition to continuous PM measurements, PM_2.5_ samples were collected to analyse their TE load. PM_2.5_ has a greater capacity for dispersion and long-range transport, making it susceptible to transfer to other carriers, which can lead to indirect pollution (He et al. 2022; WHO 2006). Table S6 summarises the average TE concentrations in PM_2.5_ for the WWTPs. At WWTP1, the average metal concentration was 12.8 ± 9.4 × 10^3^ ng m^−3^, contributing to 47.4% of the PM_2.5_ mass. The predominant elements identified in the study area were Na, Zn, K, Al, Ca, Mg, and Fe, which accounted for nearly 100% of the total metal concentration, as other elements were present at concentrations below 2 ng m^−3^. At WWTP2, the total average concentration was 7.6 ± 9.9 × 10^3^ ng m^−3^, contributing to 12.6% of the PM_2.5_ mass. The major elements identified here were also Na, Zn, K, Al, Ca, Fe, and Mg, which represented 94% of the total metal concentrations, with other elements at concentrations below 19 ng m^−3^. In all cases, the concentrations were below recommendations of the World Health Organization.

The most common sources of metals in wastewater include household products, such as detergents, body care, and cosmetics as well as industrial processes (Duan et al. 2017; Milik et al. 2017). Higher TE concentrations were found at WWTP1, likely due to its intake of water from industrial sectors, such as pharmaceuticals and galvanising factories, which could be an important source of metals (Kumar et al. 2021). Moreover, the use of superficial agitation in the bioreactor at WWTP1 could enhance TE emissions into the atmosphere through PM in the bubbles.

**Fig. 6:**
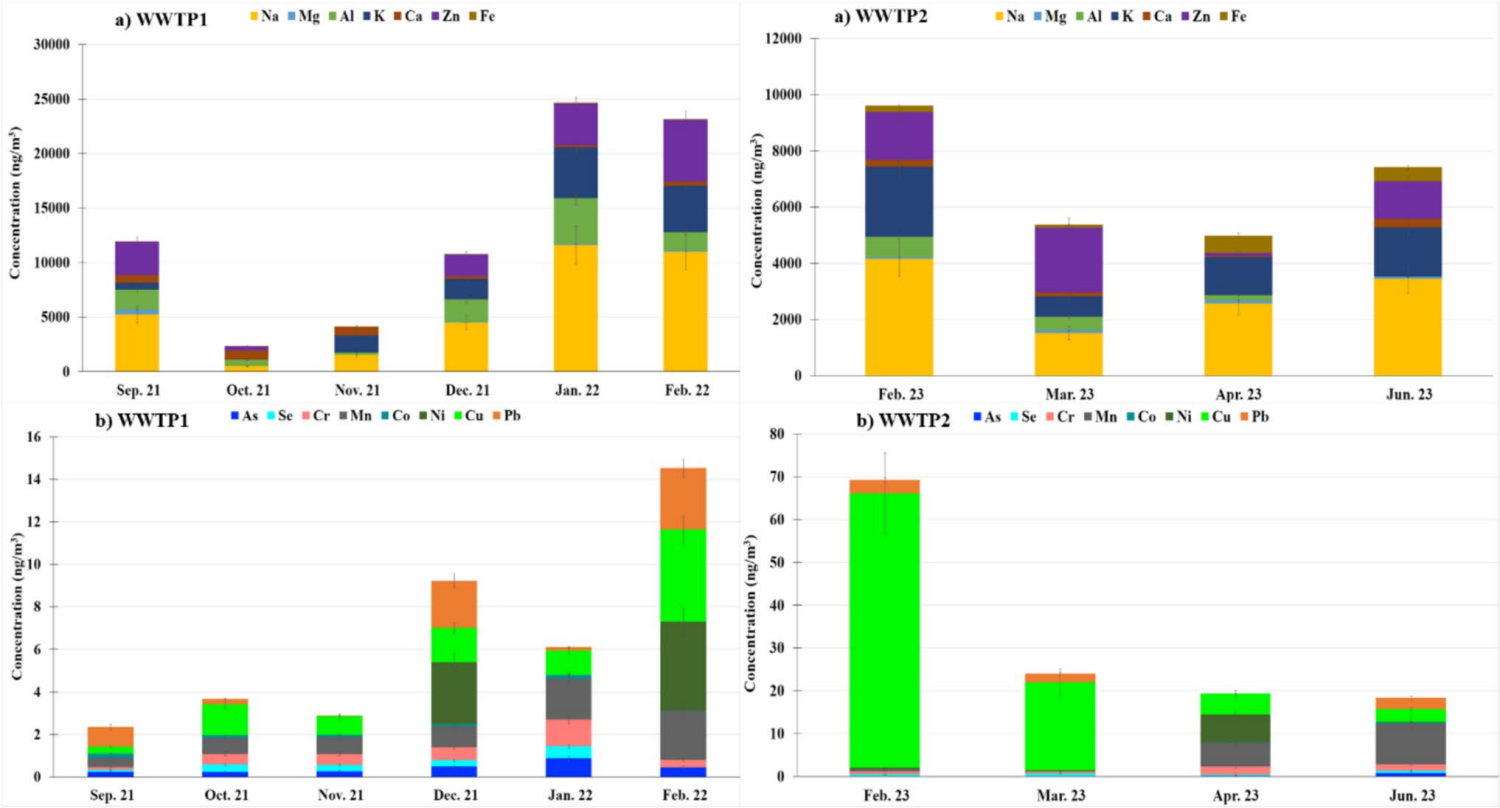
Temporal evolution of average trace element concentrations **a** majority and **b** minority.

Fig. 6 shows the seasonal evolution for TE concentrations at WWTPs. In both WWTPs, the cold season (January and February) showed higher TE levels, probably due to the lower capacity of the atmosphere to cause the mixing, dispersion, and dilution of pollutants during winter (Lei et al. 2022).

From the calculation of back trajectory frequencies during the sampling period (1 week per month), we determined the origin of the air masses, specifically on the days in which metals associated with PM_2.5_ were sampled (Fig. S10). Thus, during the sampling period, air masses from surrounding areas (30–70%) and from distant areas with lower frequency (10–20%) were obtained at both WWTPs. Far air masses came from the northwest, southeast, and Atlantic areas, whose routes passed through areas with anthropogenic emissions, such as Madrid, Valencia, and Lisbon. Moreover, to confirm the origin of the elements, the EF of each metal, considering its concentration, was calculated (Eq. S8) (Fig. S11). Thus, a low EF (< 10) was obtained for Mg, Mn, and Co at WWTP1 and for As, Cr, Na, Mg, Al, K, Ca, Mn, Co, Ni, and Pb at WWTP2. As expected, more TEs from mineral origin were detected at WWTP2; these elements probably came from erosive processes and from the suspension of mineral material of the Earth’s crust (Pey et al. 2009; Qi et al. 2016). A medium EF (10 < EF < 20) was obtained for Ca, Al, and Cr at WWTP1, indicating that their origin was hybrid. These three elements are components of the Earth’s crust (Querol et al. 2004) and are probably emitted in the metallurgical industry (Tian et al. 2016) and from WWTP (Qasem et al. 2021). High EF values (˃ 20) were obtained for Na, As, Se, K, Ni, Cu, Zn, and Pb at WWTP1 and for Zn, Cd, Cu, and Se at WWTP2, indicating an anthropogenic influence. There was a positive correlation of Ni with Cu, Pb, and Zn at WWTP1 (*p*-value < 0.05; *r* = 0.845, *r* = 0.845, and *r* = 0.676, respectively) and a significant positive correlation of Cr with Mn, Fe, and Co at WWTP2 (*p*-value < 0.05; *r* = 0.638, *r* = 0.867, and *r* = 0.875, respectively), indicating a common source, which could be the production of sludge and/or traffic (combustion and automobile tires and brake wear). Thus, these metals could proceed from the WWTP process (Karvelas et al. 2003) and, in the case of WWTP1, from nearby industry and traffic (Shabanda et al. 2019). Zn was the second major metal detected at the WWTPs (Table S6), and it had the highest EF (Fig. S11). Zn could not only come from the metallurgical industry close to WWTP1 but also from the sludge in the case of both WWTPs (Zhou et al. 2019). Moreover, in the surrounding WWTPs, the potential ecological risk (Eq. 9) showed a low Ei (< 40) for the overall elements, except for Zn at WWTP1, which had a moderate value (47.8). Therefore, according to the results obtained, the expected ecological impact is negligible.

Furthermore, exposure to TEs associated with PM can occur by ingestion, inhalation, and dermal contact, although inhalation is the most likely exposure (Zupančič et al. 2024). Once inhaled, bioavailability is the amount of the element soluble in body fluids and thus potentially available for absorption into systemic circulation. In this work, the potential health risk due to metal inhalation has been calculated from the carcinogenic and non-carcinogenic risks (Eqs. S5 and S6, respectively). For all metals, the CR values were below the reference value of 10^−6^ (Zhang et al. 2018) (Table S7), indicating that the metal concentrations did not pose a risk to staff or the nearby population. Similarly, the non-carcinogenic effects were insignificant, as all HI values were < 1 at both WWTPs during the study period (EPA 2009).

## Conclusion

This study assessed the air quality effects of two WWTPs in Toledo (Spain) by monitoring the levels of criteria pollutants (SO_2_, CO, NOx, and O_3_), VOCs, PM, and TEs. The location, quantity, and type of effluent treated, and the aeration system of the bioreactor differed. WWTP1 (located in an industrial and residential area and with mechanical aeration on the surface) treated a mix of domestic and industrial sewage and had approximately half of the flow as WWTP2 (located in the countryside area and with diffused aeration at the bottom of the bioreactor), which treated only domestic wastewater.

Higher NOx and O_3_ concentrations were observed at WWTP1, probably due to the nature of treated water and residential heating and industry close to WWTP1. For O_3_, the target threshold of 120 μg m^−3^ for human protection was reached over 27 days. Higher SO_2_ and CO concentrations were registered at WWTP2, probably due to the greater waste flow treated. These values ​did not exceed the threshold limits. Concerning VOCs, oxygenated aromatics followed by oxygenated compounds and aromatics, were the predominant compounds detected. Toluene was the top species contributing to OFP, while toluene, benzene, ethylbenzene, and xylenes were the major contributors to SOAFP at both WWTPs. Moreover, benzene showed a significant cancer risk at WWTP2, although its limit threshold value was not exceeded. Majority of the odour contributors were identified as α-pinene and aromatics, highlighting toluene at WWTP1. Therefore, covers with floating elements, such as cover balls on the surface of the bioreactor, could be an effective way to prevent or reduce evaporation and odour formation by intercepting solar radiation. For PM, the values were low and below the value threshold. For TEs in PM_2.5_, most elements identified at both WWTPs were Na, Zn, K, Al, Ca, Fe, and Mg, which primarily had a mineral origin, but others, such as Na and Zn, could come from the deodorisation process and sludge treatment at WWTP1. Overall, the potential ecological risk posed by the metals in the study area was low; thus, no adverse biological effects due to metal pollution are expected, except for Zn at WWTP1, with moderate risk. The CR values for all metals were below the reference value, indicating that the metal concentration levels did not pose a risk to staff or nearby populations. Additionally, the non-carcinogenic effects were deemed insignificant.

In summary, the results obtained at both WWTPs, despite not being critical, entailed increases in the levels of TEs, VOCs, and odours, which could cause health problems in the nearby neighbourhood or community. Therefore, WWTPs should preferably not be built near urban locations. The results may serve as a reference for other WWTPs in small cities.

## Supplementary Information

Below is the link to the electronic supplementary material.

Supplementary file1 (DOCX 19310 KB)

## Data Availability

Data and materials would be available on reasonable request.
