# Supplementary material for: Assessment of airborne pollutants in wastewater treatment plants
Source: Environ Sci Pollut Res Int. 2025 May 7;32(21):12868–82. doi: 10.1007/s11356-025-36484-3 (PMC12119708; doi:10.1007/s11356-025-36484-3)
Supplement: Supplementary file 1 — Supplementary file1 (DOCX 19310 KB) [file 11356_2025_36484_MOESM1_ESM.docx]

**Assessment of Airborne Pollutants in Wastewater Treatment Plants**

Gabriela Viteri^a^, Alfonso Aranda^a^, Yolanda Díaz-de-Mera^a^, Ana Rodríguez^b^, Nuria Rodriguez-Fariñas^b^, Diana Rodríguez^b^*

^a^Universidad de Castilla-La Mancha, Facultad de Ciencias y Tecnologías Químicas, Departamento de Química Física, Avda. Camilo José Cela 1B, 13071, Ciudad Real, Spain.

^b^Universidad de Castilla-La Mancha, Facultad de Ciencias Ambientales y Bioquímica, Departamento de Química Física, Avenida Carlos III s/n, 45071, Toledo, Spain.

*Corresponding author: diana.rodriguez@uclm.es, (34)926051472

**Fig. S1**.

Map showing the geographical locations of the wastewater treatment plants (WWTPs), the air quality station in Toledo, and the surrounding areas, along with a plan of the sewage treatment facilities. The red star indicates the position of the sampling point

**Text S1. Chemical analysis by the ICP-MS**

A triple quadrupole ICP-MS iCap-TQ (Thermo Electron Corporation, Germany) equipped with a Micromist nebulizer, and a cyclonic spray chamber was employed for total concentration analysis. Operational ICP-MS conditions are summarized in Table S1. The equipment was tuned daily for the highest sensitivity and worked in the Single Quadrupole Kinetic Energy Discrimination mode (SQ-KED) with He as collision and focussing gas as standard mode for spectral and polyatomic interference removal. The raw data of the isotope signals for the different elements (Table S1) and rhodium (15 μg L^-1^, internal standard used to correct signal drift) were further processed using the Qtegra™ Intelligent Scientific Data Solution™ (ISDS) software for total analysis.

Solutions used for calibration were prepared from commercial stock standards with 1 g/L of each element (Inorganic Ventures, Virginia, USA). The relative signal ratio (each isotope over Rh) was converted to concentration using an external calibration curve for each element (0.2–100 μg L^-1^). The detection limits (LODs, in μg/g, back calculated to 0.025 g in filter sample) and metal concentrations (μg/ g) in the blank filter sample are shown in Table S2. Furthermore, considering all the procedures for TE analysis, an uncertainty of around 15% of the measurement value was estimated.

Also, quality controls were performed. On the one hand, the intensity of Rh internal standard for every sample analysis and the full calibration curve and blanks were run at a frequency of every 20 samples. On the other hand, the accuracy of the methodology was evaluated by analyzing a control filter supplemented with a standard solution of Pb, As, Ni and Cd in concentration of 10 μg/ L. The recovery for the determination of these metals were close to 100% in all cases.

**Table S1.** ICP-MS operating conditions

| RF-Power (KW) | 1.5 |
| --- | --- |
| Plasma gas flow rate (L min^-1^) | 14 |
| Carrier gas flow rate (L min^-1^) | 1 |
| Nebulizer flow rate (L min^-1^) | 0.8 |
| Auxiliary gas flow rate (L min^-1^) | 0.8 |
| Spray chamber | Cyclonic type |
| Isotopes monitored | ^23^Na, ^24^Mg, ^27^Al, ^39^K, ^44^Ca, ^52^Cr, ^55^Mn, ^57^Fe, ^59^Co, ^60^Ni, ^63^Cu, ^66^Zn, ^75^As, ^78^Se, ^103^Rh, ^111^Cd, ^202^Pt, ^208^Pb |
| Mode | SQ-KED |
| He flow rate (mL min^-1^) | 4.9 |
| Q1 Bias (V) | -2.5 |
| QCell Bias (V) | -2.0 |
| Q3 Bias (V) | -1.0 |
| Dwell time (ms) | 10 |

**Table S2**. The detection limits and concentration in blank filter sample.

| **Trace Element** | **LOD**  **(ug/g)** | **Blank filter sample (ug/g)** |
| --- | --- | --- |
| Na | 14.8 | 42,580 |
| Zn | 0.99 | 17,900 |
| K | 1,42 | 18,030 |
| Al | 8.6 | 10,100 |
| Ca | 6.1 | 3,100 |
| Mg | 6.5 | 1,140 |
| Fe | 6.0 | 450 |
| Cu | 0.36 | 2.2 |
| Mn | 0.14 | 21 |
| Ni | 0.24 | ND |
| Pb | 0.24 | 25.0 |
| Cr | 0.8 | 5.5 |
| As | 0.006 | 23.0 |
| Se | 0.03 | 1.6 |
| Co | 0.006 | 1.0 |
| Cd | 0.02 | 3.3 |
| Hg | 0.2 | 0.1 |

**Text S2. Environmental and health risk indicators**

a) OFP assesses the contribution of VOC species to formation O_3_.

OFP_i_ =[VOC_i_]×MIR_i_ (Equation 1)

where: [VOC_i_] is the concentration (µg m^-3^) of the VOC species i; MIR_i_ is the maximum incremental reactivity of VOC_i_ (Carter, 2010).

b) SOAP reflect the tendency of each VOC species i to form SOA on the same mass emission basis as toluene (Derwent et al., 2010):

$P_{{SOAFP}_{i}=\frac{{VOC}_{i} \times{SOAP}_{i}}{100} \times{FAC}_{toluene}}$ (Equation 2)

where VOC_i_ is the concentration (µg m^-3^) of the VOC species i; FAC_toluene_ is the fractional aerosol coefficient of toluene (5.4%, according to the value defined in Zhang et al., 2017), and SOAP_i_ is the potential of a VOC species to form SOA relative to the same mass of toluene when it is added to atmosphere:

${SOAP}_{i}=\frac{Increment in SOA mass concentration with species}{Increment in SOA with toluene}\times100$ (Equation 3)

c) CR of elements and VOCs entering the body by inhalation:

$CR= {ADDE}_{inh} \times CSF$ (Equation 4)

where ADDE_inh_ is the average daily dose exposure of the inhaled pollutant and CSF is the carcinogenic factor of the pollutant (Singh et al., 2016). The ADDE_inh_ was calculated from Equation 5:

${ADDE}_{inh}= \frac{C\times IR\times EF\times ED}{BW\times AT}$ (Equation 5)

where C is the average concentration for each pollutant (mg m^-3^), IR is the inhalation rate for an adult (20 m^3^ per day), EF is the exposure frequency (365 days per year), ED is the exposure duration (30 years), BW is the body weight for an adult (70 kg), and AT is the average time (i.e. 70 years × 365 days/year) (Widiana, et al., 2019; Chen, et al., 2022).

d)Whereas the *non-carcinogenic risk* was determined in terms of the hazard index (HI) which is defined as the ratio of ADDE_inh_ to the reference dose (RfD) (Widiana et al., 2019).

$HI= \frac{{ADDE}_{ihn}}{RfD}$ (Equation 6)

where RfD refers to an estimated level of human daily intake without adverse health effects during a lifetime (mg kg^-1^ × day) and can be estimated from the corresponding value of the reference concentration (RfC) by Equation 7.

$RfD= \frac{RfC \times IR}{BW}$ (Equation 7)

HI can be classified in the following order: values less than 1.0 show a low hazard risk; HI values in the range of 1.1-10 show a moderate hazard risk, and those over 10 show a high hazard risk (EPA, 2009).

e) EF ratio denotes the degree of enrichment of a particular element when compared with the relative abundance of that element in the Earth’s crust (Watson et al., 2002). This comparison is usually carried out with respect to an abundant crustal element. We opted for Fe due to its high natural abundance and relatively small influence of anthropogenic sources (Adimalla et al., 2019; Jiang et al., 2020).

EF=(B_i_ /B_Fe_ )(C_i_ /C_Fe_ ) (Equation 8)

where C_i_ is the concentration of trace elements, C_Fe_ is the concentration of Fe in the sample, B_i_ is the background value of the interest element, and B_Fe_ is the background value of Fe. The EFs are analyzed using the two-threshold method, where EF < 10 indicates a relevant crustal contribution, EF > 20 suggests a likely anthropogenic origin, and 10 < EF < 20 indicates a mixed origin with contributions from both natural and anthropogenic sources.

f) RI assesses the ecological risk of heavy metals in sediments by considering metal toxicity and comparing metal concentrations to background values (Hakanson, 1980):

$RI=\sum_{i}^{n} E_{i}=\sum_{i}^{n} T_{i}\times C_{f}^{i}=\sum_{i}^{n} T_{i}\times\frac{C_{i}}{B_{i}}$ (Equation 9)

where *E_i_* is the potential ecological risk factor of metal *i*. *T_i_* is the metal toxic factor, i.e., Hg = 40, Cd=30, As = 10, Ni=Cu = Pb = Co= 5, Cr = 2, Zn = Mn = 1 and Fe= 0 (Hakanson, 1980; Xu et al., 2008). C_ƒ_*^i^* is the metal pollution factor of metal *i*, which equals the amount of metal *i* in the sample (*C_i_*) divided by its reference value (*B_i_*).

RI can be classified in the following order: low ecological risk (*Ei*<40, *RI*<90), moderate ecological risk (40≤*Ei*<80, 90≤*RI*<180), considerable ecological risk (80≤*Ei*<160, 180≤*RI*<360), high ecological risk (160≤*Ei*<320, 320≤*RI*<720) and very high ecological risk (*Ei*≥320, *RI*≥720) (Al-shidi et al., 2021).

**References:**

Adimalla N, Qian H, Wang H (2019) Assessment of heavy metal (HM) contamination in agricultural soil lands in northern Telangana, India: an approach of spatial distribution and multivariate statistical analysis. Environ Monit Assess 191: 246. <https://doi.org/10.1007/s10661-019-7408-1>

Al-Shidi HK, Sulaiman H, Al-Reasi HA, Jamil F, Aslam M (2021) Human and ecological risk assessment of heavy metals in different particle sizes of road dust in Muscat, Oman. Environ Sci Pollut Res 28: 33980-33993. <https://doi.org/10.1007/s11356-020-09319-6>

Chen X, Ward TJ, Sarkar C, Ho K, Webster C (2022) Health risks of adults in Hong Kong related to inhalation of particle-bound heavy metal(loid)s. Air Qual Atmos Health. 15: 691-706. <https://doi.org/10.1007/s11869-021-01115-6>

Derwent RG, Jenkin ME, Utembe SR, Shallcross DE, Murrells TP, Passant NR (2010) Secondary organic aerosol formation from a large number of reactive manmade organic compounds. Sci Total Environ. 408: 3374-3381. <https://doi.org/10.1016/j.scitotenv.2010.04.013>

Hakanson L (1980) An ecological risk index for aquatic pollution control: a sedimentological approach. Water Res 14: 975-1001. <https://doi.org/10.1016/0043-1354(80)90143-8>

Jiang HH, Cai LM, Wen HH. Hu GC, Chen LG, Luo J (2020) An integrated approach to quantifying ecological and human health risks from different sources of soil heavy metals. Sci Total Environ 701: 134466. <https://doi.org/10.1016/j.scitotenv.2019.134466>

Singh D, Kumar A, Kumar K, Singh B, Mina U, Singh BB, Jain VK (2016) Statistical modelling of O_3_, NOx, CO, PM_2.5_, VOCs and noise levels in commercial complex and associated health risk assessment in an academic institution. Sci Total Environ 572: 586-594. <https://doi.org/10.1016/j.scitotenv.2016.08.086>

Watson JG, Zhu T, Chow JC, Engelbrecht J, Fujita EM, Wilson WE (2002) Receptor modelling application framework for particle source apportionment. Chemosphere 49: 1093-1136. <https://doi.org/10.1016/S0045-6535(02)00243-6>

Widiana DR, Wang Y-F, You S-J, Yang HH, Wang LC, Tsai J-H, Chen HM (2019) Air Pollution Profiles and Health Risk Assessment of Ambient Volatile Organic Compounds above a Municipal Wastewater Treatment Plant, Taiwan. Aerosol and Air Quality Research 19: 375-382. <https://doi.org/10.4209/aaqr.2018.11.0408>

Xu ZQ, Ni SJ, Tuo XG, Zhang CJ (2008) Calculation of Heavy Metals’ Toxicity Coefficient in the Evaluation of Potential Ecological Risk Index. Environ Sci Technol 31: 112-115.

Zhang Z, Wang H, Chen D, Li Q, Thai P, Gong D, Li Y, Zhang C, Gu Y, Zhou L, Morawska L, Wang B (2017) Emission characteristics of volatile organic compounds and their secondary organic aerosol formation potentials from a petroleum refinery in Pearl River Delta, China. Sci Total Environ 584: 1162-1174. <https://doi.org/10.1016/j.scitotenv.2017.01.179>


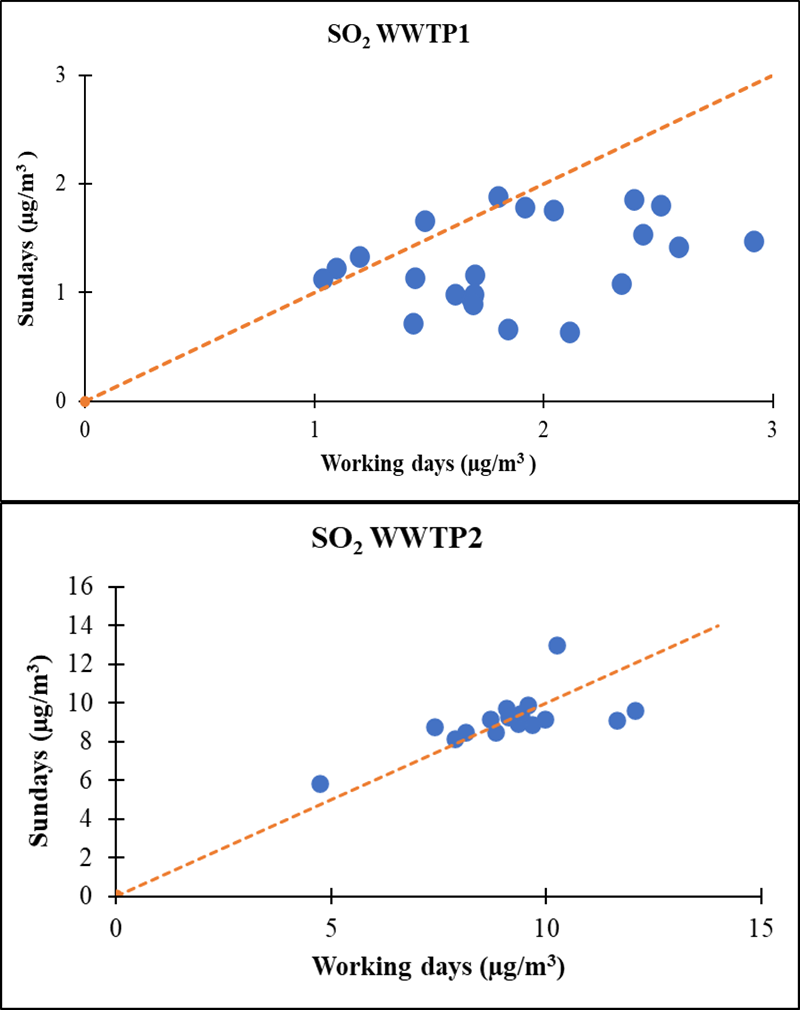


**Fig. S2.** Weekly average SO_2_ concentrations: Sundays vs working days (Monday-Saturday).


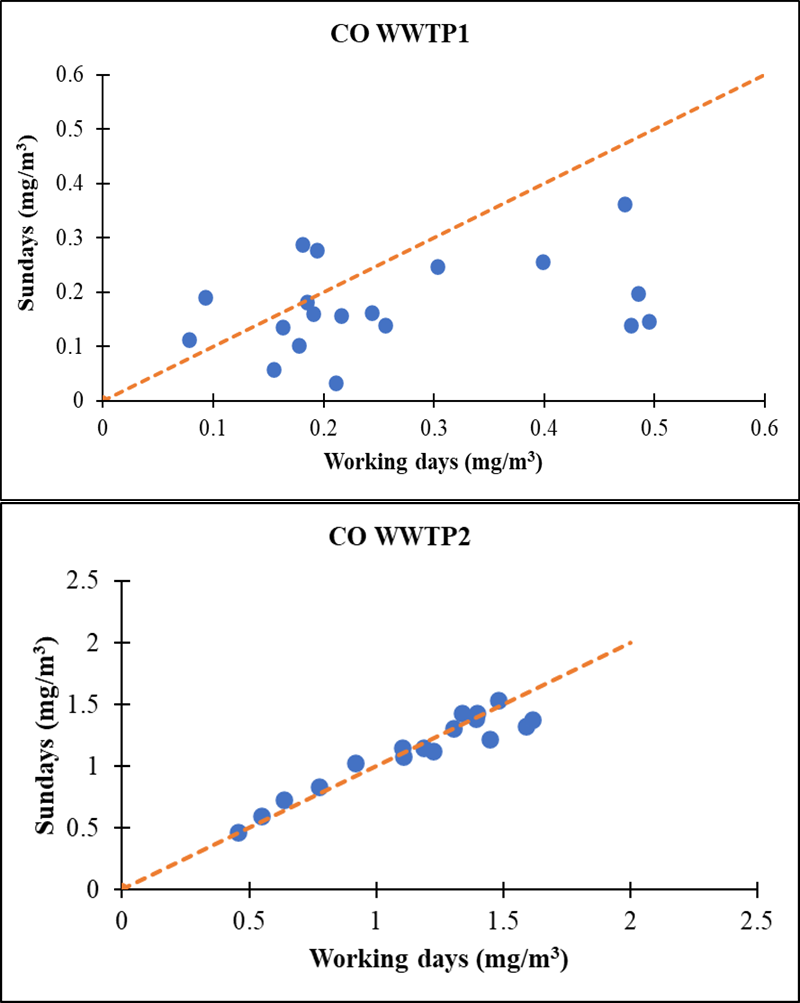


**Fig. S3**. Weekly average CO concentrations: Sundays vs working days (Monday-Saturday).


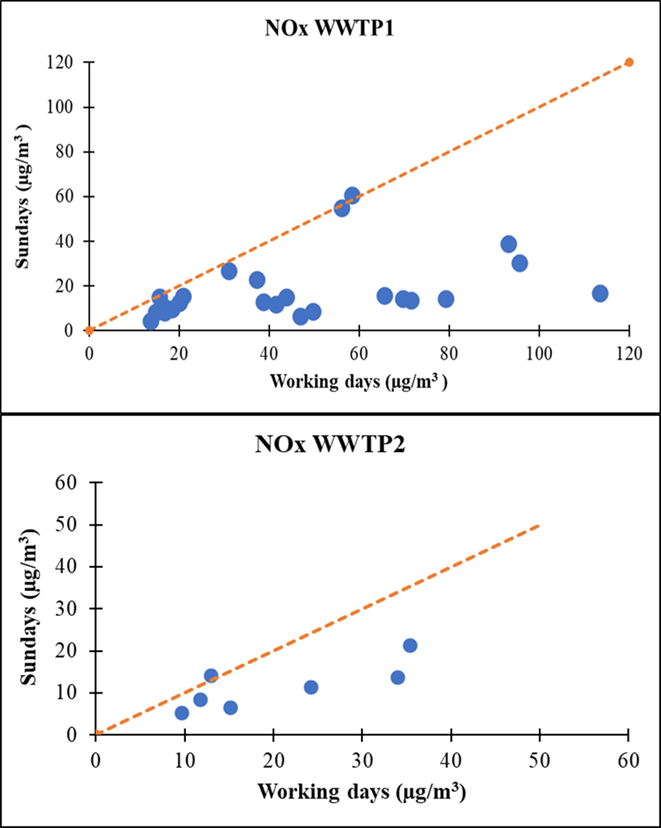


**Fig. S4.** Weekly average NOx concentrations: Sundays vs working days (Monday-Saturday).


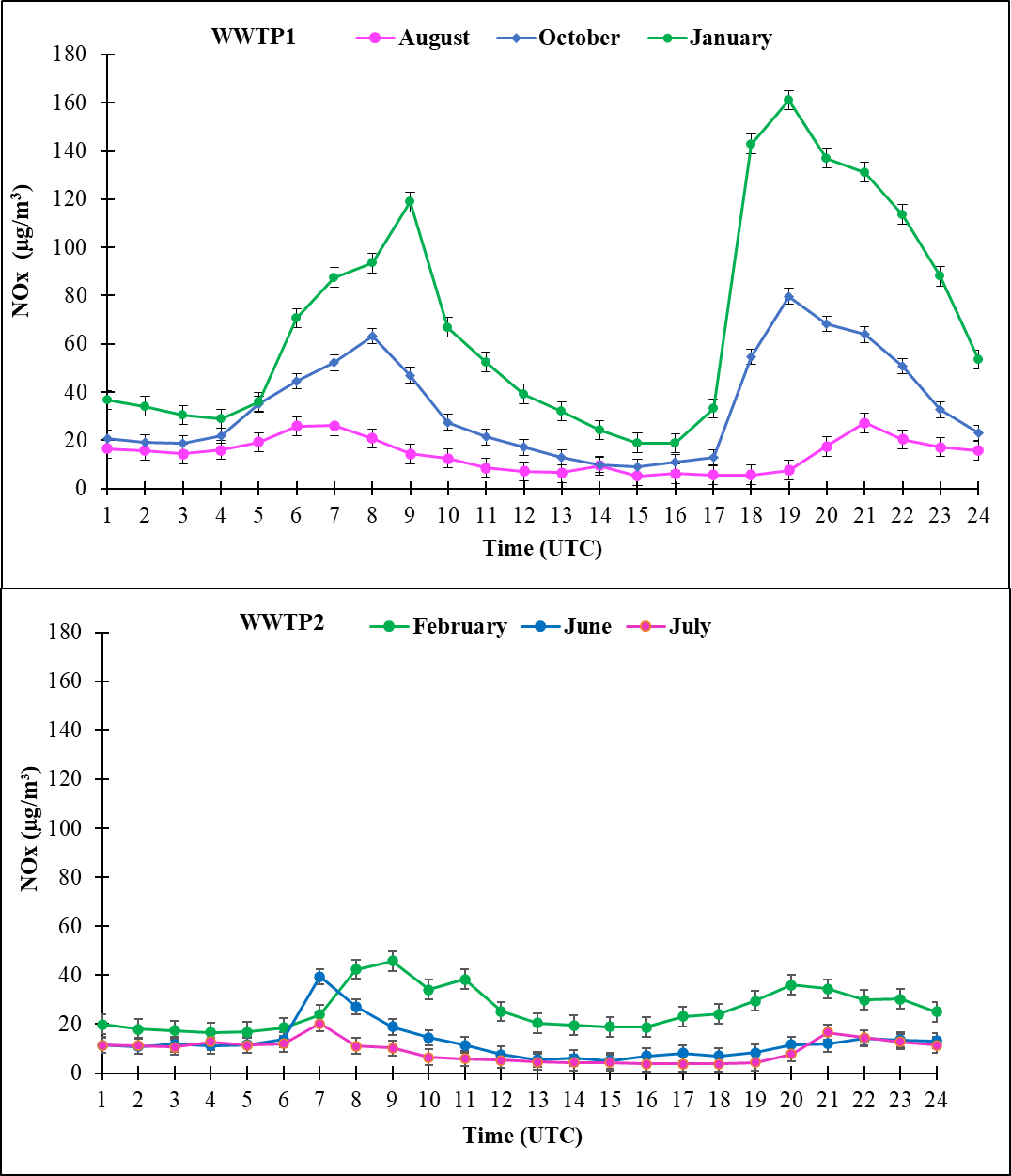


**Fig. S5**. Hourly average daily profiles of NOx in both WWTPs.


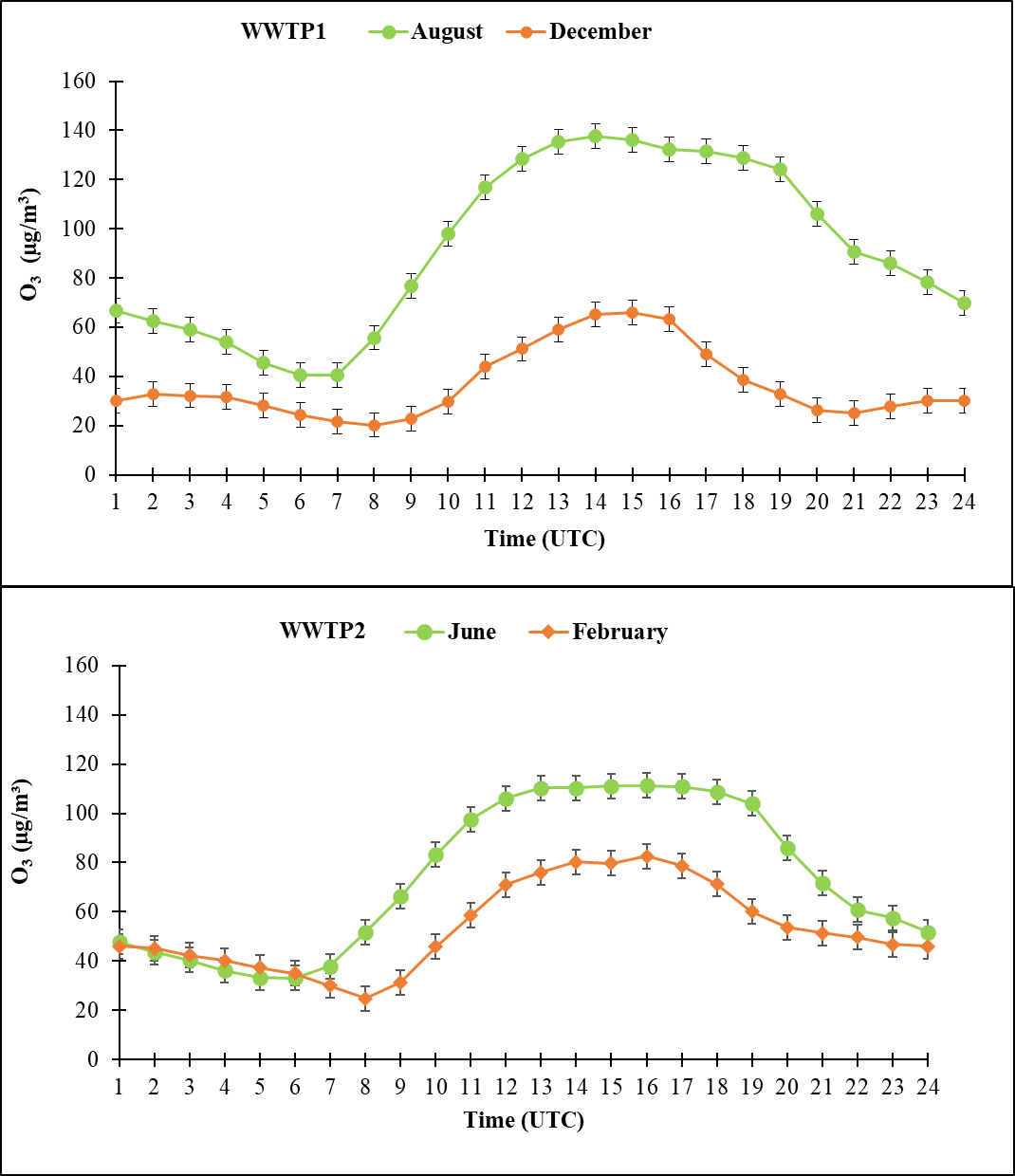


**Fig. S6**. Hourly average daily profiles of O_3_ in both WWTPs.

**Table S3**. Average concentrations for the four studied timeframes, maximum, minimum, and overall average concentrations observed for the different compounds during the campaigns. Max and min are the values of the campaigns with the highest and lowest concentration, respectively. Units in μg m^-3^.

| **WWTP1** | **12:00-18:00** | **18:00-0:00** | **0:00-6:00** | **06:00-12:00** | **Max** | **Min** | **Average** | **WWTP2** | **12:00 - 18:00** | **18:00 - 0:00** | **0:00 - 06:00** | **06:00 - 12:00** | **Max** | **Min** | **Average** |
| --- | --- | --- | --- | --- | --- | --- | --- | --- | --- | --- | --- | --- | --- | --- | --- |
| **Benzoic acid** | 2.62E+01 | 6.38E+00 | 2.61E+01 | 6.10E+01 | 6.10E+01 | 6.38E+00 | 2.55E+01 | **Benzoic acid** | 1.23E+01 | 1.35E+01 | 2.66E+01 | 1.89E+01 | 2.66E+01 | 1.23E+01 | 1.78E+01 |
| **Toluene** | 6.70E+00 | 1.01E+01 | 2.67E+01 | 2.90E+01 | 3.38E+01 | 3.99E+00 | 1.89E+01 | **Acetophenone** | 1.12E+01 | 9.91E+00 | 1.56E+01 | 9.41E+00 | 1.56E+01 | 9.41E+00 | 1.15E+01 |
| **Acetone** | 3.60E+00 | 1.42E+01 | 4.62E+00 | 4.57E+00 | 1.42E+01 | 3.60E+00 | 6.75E+00 | **Decanal** | 7.73E+00 | 5.82E+00 | 8.88E+00 | 3.62E+00 | 8.88E+00 | 3.62E+00 | 6.51E+00 |
| **Methyl Acetate** | 4.90E+00 | 1.95E+00 | 2.14E+00 | 2.06E+00 | 4.90E+00 | 1.95E+00 | 2.76E+00 | **Nonanal** | 6.86E+00 | 4.45E+00 | 7.27E+00 | 3.30E+00 | 7.27E+00 | 3.30E+00 | 5.47E+00 |
| **Octanoic acid** | 6.50E-01 | 6.78E+00 | 1.73E+00 | 1.65E+00 | 6.78E+00 | 6.50E-01 | 2.70E+00 | **Benzaldehyde** | 5.02E+00 | 4.03E+00 | 7.17E+00 | 3.85E+00 | 7.17E+00 | 3.85E+00 | 5.02E+00 |
| **Butyl Acetate** | 4.87E-01 | 3.63E+00 | 3.69E+00 | 1.30E+00 | 3.69E+00 | 4.87E-01 | 2.28E+00 | **Undecanal** | 4.65E+00 | 3.69E+00 | 2.39E+00 | 7.81E+00 | 7.81E+00 | 2.39E+00 | 4.64E+00 |
| **α-pinene** | 1.72E+00 | 2.97E+00 | 1.52E+00 | 1.71E+00 | 2.97E+00 | 1.52E+00 | 1.98E+00 | **2-Butanone** | 4.73E+00 | 5.89E+00 | 1.69E+00 | 2.20E+00 | 5.89E+00 | 1.69E+00 | 3.63E+00 |
| **m-p Xylene** | 6.86E-01 | 1.65E+00 | 2.30E+00 | 1.84E+00 | 2.04E+00 | 1.03E+00 | 1.53E+00 | **Benzene** | 4.15E+00 | 2.88E+00 | 5.07E+00 | 1.65E+00 | 5.07E+00 | 1.65E+00 | 3.44E+00 |
| **Ethyl Acetate** | 2.09E+00 | 1.40E+00 | 5.96E-01 | 8.12E-01 | 2.09E+00 | 5.96E-01 | 1.22E+00 | **Ethyl Acetate** | 3.95E+00 | 6.52E+00 | 1.09E+00 | 1.16E+00 | 6.52E+00 | 1.09E+00 | 3.18E+00 |
| **Benzene** | 7.88E-01 | 8.51E-01 | 9.66E-01 | 9.87E-01 | 9.06E-01 | 7.48E-01 | 8.27E-01 | **Toluene** | 2.62E+00 | 2.19E+00 | 4.91E+00 | 2.81E+00 | 4.91E+00 | 2.19E+00 | 3.13E+00 |
| **Acetophenone** | 5.81E-01 | 9.82E-01 | 8.91E-01 | 5.51E-01 | 9.82E-01 | 5.51E-01 | 7.51E-01 | **Phenol** | 2.05E+00 | 2.06E+00 | 4.61E+00 | 1.89E+00 | 4.61E+00 | 1.89E+00 | 2.65E+00 |
| **1,1,2-trichlorotrifluoroethane** | 9.68E-01 | 7.10E-01 | 7.97E-01 | 8.87E-01 | 7.55E-01 | 7.10E-01 | 7.32E-01 | **Carbon tetrachloride** | 2.19E+00 | 1.39E+00 | 1.39E+00 | 1.30E+00 | 2.19E+00 | 1.30E+00 | 1.57E+00 |
| **o- Xylene** | 2.77E-01 | 6.16E-01 | 8.37E-01 | 7.29E-01 | 7.22E-01 | 4.52E-01 | 5.87E-01 | **Dodecanal** | 2.35E+00 | 1.31E+00 | 1.39E+00 | 7.17E-01 | 2.35E+00 | 7.17E-01 | 1.44E+00 |
| **2-Butanone** | 4.44E-01 | 8.00E-01 | 3.89E-01 | 3.01E-01 | 8.00E-01 | 3.01E-01 | 4.84E-01 | **Octanal** | 1.44E+00 | 8.97E-01 | 1.80E+00 | 7.26E-01 | 1.80E+00 | 7.26E-01 | 1.22E+00 |
| **Nonane** | 2.54E-01 | 9.18E-01 | 4.54E-01 | 2.90E-01 | 9.18E-01 | 2.54E-01 | 4.79E-01 | **Heptanal** | 1.15E+00 | 5.25E-01 | 1.40E+00 | 6.20E-01 | 1.40E+00 | 5.25E-01 | 9.24E-01 |
| **Ethylbenzene** | 2.40E-01 | 4.71E-01 | 6.75E-01 | 5.71E-01 | 5.93E-01 | 3.40E-01 | 4.67E-01 | **m-p Xylene** | 4.20E-01 | 4.66E-01 | 1.38E+00 | 1.30E+00 | 1.38E+00 | 4.20E-01 | 8.90E-01 |
| **Decane** | 1.64E-01 | 8.17E-01 | 5.19E-01 | 2.48E-01 | 8.17E-01 | 1.64E-01 | 4.37E-01 | **Chloroform** | 7.66E-01 | 4.87E-01 | 1.73E+00 | 4.14E-01 | 1.73E+00 | 4.14E-01 | 8.49E-01 |
| **Carbon tetrachloride** | 4.76E-01 | 4.39E-01 | 3.85E-01 | 3.66E-01 | 4.21E-01 | 3.67E-01 | 3.94E-01 | **Hexanal** | 7.38E-01 | 3.58E-01 | 8.26E-01 | 2.93E-01 | 8.26E-01 | 2.93E-01 | 5.54E-01 |
| **n-Hexane** | 2.65E-01 | 3.77E-01 | 5.00E-01 | 4.35E-01 | 5.99E-01 | 1.80E-01 | 3.90E-01 | **Nonane** | 6.89E-01 | 6.02E-01 | 3.36E-01 | 2.19E-01 | 6.89E-01 | 2.19E-01 | 4.62E-01 |
| **Styrene** | 7.70E-02 | 4.24E-01 | 4.16E-01 | 2.63E-01 | 4.31E-01 | 2.18E-01 | 3.24E-01 | **Decane** | 7.78E-01 | 5.78E-01 | 2.12E-01 | 1.72E-01 | 7.78E-01 | 1.72E-01 | 4.35E-01 |
| **n-Heptane** | 1.75E-01 | 2.54E-01 | 4.26E-01 | 3.61E-01 | 3.09E-01 | 2.66E-01 | 2.88E-01 | **Undecane** | 8.09E-01 | 4.64E-01 | 1.47E-01 | 1.79E-01 | 8.09E-01 | 1.47E-01 | 4.00E-01 |
| **1,2,4-trimethylbenzene** | 1.38E-01 | 3.10E-01 | 3.70E-01 | 3.24E-01 | 3.59E-01 | 1.77E-01 | 2.68E-01 | **Ethylbenzene** |  | 1.69E-01 | 5.80E-01 | 4.35E-01 | 5.80E-01 | 1.69E-01 | 3.95E-01 |
| **Undecanal** | 1.59E-01 | 4.59E-01 | 2.23E-01 | 1.44E-01 | 4.59E-01 | 1.44E-01 | 2.46E-01 | **o-Xylene** | 1.82E-01 | 1.91E-01 | 5.14E-01 | 4.70E-01 | 5.14E-01 | 1.82E-01 | 3.39E-01 |
| **1-Butanol** | 1.74E-01 | 1.74E-01 | 1.74E-01 | 1.74E-01 | 1.74E-01 | 1.74E-01 | 1.74E-01 | **alpha.pinene** | 5.53E-01 | 3.19E-01 | 1.96E-01 | 2.81E-01 | 5.53E-01 | 1.96E-01 | 3.37E-01 |
| **Undecane** | 1.21E-01 | 2.94E-01 | 1.49E-01 | 1.06E-01 | 2.94E-01 | 1.06E-01 | 1.67E-01 | **Dodecane** | 3.64E-01 | 3.17E-01 | 3.70E-01 | 1.63E-01 | 3.70E-01 | 1.63E-01 | 3.03E-01 |
| **2-nitropropane** | 1.79E-01 | 1.20E-01 | 2.12E-01 | 3.04E-01 | 2.94E-01 | 2.83E-02 | 1.61E-01 | **1-Butanol** | 2.12E-01 | 4.51E-01 | 2.79E-01 | 2.02E-01 | 4.51E-01 | 2.02E-01 | 2.86E-01 |
| **Chloroform** | 1.35E-01 | 1.43E-01 | 2.24E-01 | 2.16E-01 | 1.62E-01 | 1.46E-01 | 1.54E-01 | **1,2-dichloroethane** | 3.24E-01 | 2.86E-01 | 2.13E-01 | 1.91E-01 | 3.24E-01 | 1.91E-01 | 2.54E-01 |
| **n-Octane** | 8.21E-02 | 1.71E-01 | 1.60E-01 | 1.84E-01 | 1.76E-01 | 1.19E-01 | 1.48E-01 | **Pentanal** | 3.47E-01 | 1.90E-01 | 2.54E-01 | 1.17E-01 | 3.47E-01 | 1.17E-01 | 2.27E-01 |
| **Dodecane** | 5.85E-02 | 1.53E-01 | 2.01E-01 | 1.01E-01 | 2.01E-01 | 5.85E-02 | 1.28E-01 | **p-Cymene** | 4.74E-02 | 1.31E-01 | 3.15E-01 | 2.63E-01 | 3.15E-01 | 4.74E-02 | 1.89E-01 |
| **p-Cymene** | 4.34E-02 | 1.11E-01 | 2.19E-01 | 1.22E-01 | 1.62E-01 | 5.50E-02 | 1.08E-01 | **Tetrachloroethene** | 1.58E-01 | 1.33E-01 | 2.53E-01 | 1.78E-01 | 2.53E-01 | 1.33E-01 | 1.80E-01 |
| **n-propylbenzene** | 4.68E-02 | 1.16E-01 | 1.15E-01 | 9.29E-02 | 1.37E-01 | 4.00E-02 | 8.87E-02 | **Tetrahydrofuran** | 9.79E-02 | 8.43E-02 | 3.28E-01 | 7.39E-02 | 3.28E-01 | 7.39E-02 | 1.46E-01 |
| **Tetrahydrofuran** | 4.20E-02 | 6.32E-02 | 2.12E-01 | 1.13E-01 | 1.03E-01 | 7.19E-02 | 8.75E-02 | **n-Octane** | 1.36E-01 | 1.40E-01 | 1.54E-01 | 9.52E-02 | 1.54E-01 | 9.52E-02 | 1.31E-01 |
| **Tetrachloroethene** | 4.60E-02 | 7.60E-02 | 1.39E-01 | 9.50E-02 | 8.95E-02 | 7.59E-02 | 8.27E-02 | **1,2,4-trimethylbenzene** | 7.10E-02 | 8.34E-02 | 1.63E-01 | 1.25E-01 | 1.63E-01 | 7.10E-02 | 1.11E-01 |
| **1,3,5-trimethylbenzene** | 4.31E-02 | 9.09E-02 | 1.05E-01 | 1.01E-01 | 1.08E-01 | 5.06E-02 | 7.93E-02 | **n-Heptane** | 1.81E-01 | 9.65E-02 | 7.95E-02 | 6.95E-02 | 1.81E-01 | 6.95E-02 | 1.07E-01 |
| **Napthalene** | 3.35E-02 | 8.62E-02 | 7.95E-02 | 7.26E-02 | 9.23E-02 | 4.40E-02 | 6.82E-02 | **Styrene** | 9.33E-02 | 1.05E-01 | 9.01E-02 | 7.26E-02 | 1.05E-01 | 7.26E-02 | 9.02E-02 |
| **4-Ethyltolueno** | 2.88E-02 | 6.23E-02 | 8.45E-02 | 6.94E-02 | 8.99E-02 | 2.89E-02 | 5.94E-02 | **Bromoform** | 9.61E-02 | 7.37E-02 | 8.78E-02 | 7.91E-02 | 9.61E-02 | 7.37E-02 | 8.42E-02 |
| **2-Ethyltoluene** | 3.06E-02 | 6.09E-02 | 7.32E-02 | 6.81E-02 | 7.72E-02 | 3.23E-02 | 5.47E-02 | **Naphthalene** | 9.32E-02 | 7.93E-02 | 8.72E-02 | 6.34E-02 | 9.32E-02 | 6.34E-02 | 8.08E-02 |
| **1,2,3-trimethylbenzene** | 2.36E-02 | 5.71E-02 | 6.99E-02 | 6.35E-02 | 6.89E-02 | 3.03E-02 | 4.96E-02 | **1,2-dichloropropane** | 7.34E-02 | 6.43E-02 | 7.19E-02 | 5.53E-02 | 7.34E-02 | 5.53E-02 | 6.62E-02 |
| **Isopropylbenzene** | 1.98E-02 | 3.55E-02 | 4.29E-02 | 3.51E-02 | 4.02E-02 | 2.39E-02 | 3.21E-02 | **Camphene** | 1.07E-01 | 6.25E-02 | 3.02E-02 | 5.50E-02 | 1.07E-01 | 3.02E-02 | 6.37E-02 |
| **Bromoform** | 2.03E-02 | 2.31E-02 | 2.70E-02 | 2.05E-02 | 3.36E-02 | 1.40E-02 | 2.38E-02 | **Isopropylbenzene** | 5.25E-02 | 5.48E-02 | 7.96E-02 | 5.55E-02 | 7.96E-02 | 5.25E-02 | 6.06E-02 |
| **1,2-dichloropropane** | 1.69E-02 | 1.76E-02 | 1.82E-02 | 1.65E-02 | 1.94E-02 | 1.46E-02 | 1.70E-02 | **Dibromomethane** | 6.26E-02 | 4.73E-02 | 5.06E-02 | 3.72E-02 | 6.26E-02 | 3.72E-02 | 4.94E-02 |
| **Bromodichloromethane** | 1.14E-02 | 1.51E-02 | 1.79E-02 | 1.47E-02 | 1.67E-02 | 1.14E-02 | 1.40E-02 | **2-Ethyltoluene** | 3.52E-02 | 3.91E-02 | 6.61E-02 | 5.18E-02 | 6.61E-02 | 3.52E-02 | 4.81E-02 |
| **Propionitrile** | 1.22E-02 | 1.01E-02 | 1.06E-02 | 1.42E-02 | 1.16E-02 | 9.68E-03 | 1.07E-02 | **Bromodichloromethane** | 4.43E-02 | 4.27E-02 | 5.71E-02 | 3.86E-02 | 5.71E-02 | 3.86E-02 | 4.57E-02 |
| **Chlorobenzene** | 7.17E-03 | 1.16E-02 | 1.03E-02 | 9.80E-03 | 1.44E-02 | 5.63E-03 | 1.00E-02 | **Chlorobenzene** | 3.50E-02 | 4.75E-02 | 7.28E-02 | 2.09E-02 | 7.28E-02 | 2.09E-02 | 4.41E-02 |
| **n-butylbenzene** | 5.00E-03 | 1.00E-02 | 1.29E-02 | 1.29E-02 | 1.27E-02 | 6.84E-03 | 9.77E-03 | **1,1,1-trichloroethane** | 5.06E-02 | 3.81E-02 | 4.51E-02 | 3.57E-02 | 5.06E-02 | 3.57E-02 | 4.24E-02 |
| **Dibromochloromethane** | 8.32E-03 | 1.01E-02 | 1.15E-02 | 8.91E-03 | 1.16E-02 | 7.52E-03 | 9.53E-03 | **Trichloroethene** | 3.65E-02 | 6.09E-02 | 3.08E-02 | 2.29E-02 | 6.09E-02 | 2.29E-02 | 3.78E-02 |
| **sec-butylbenzene** | 6.27E-03 | 9.37E-03 | 1.04E-02 | 1.03E-02 | 9.63E-03 | 7.84E-03 | 8.73E-03 | **n-propylbenzene** | 3.32E-02 | 2.56E-02 | 5.65E-02 | 3.44E-02 | 5.65E-02 | 2.56E-02 | 3.74E-02 |
| **Dibromomethane** | 8.00E-03 | 8.76E-03 | 9.40E-03 | 8.42E-03 | 9.26E-03 | 7.10E-03 | 8.18E-03 | **1,4-dichlorobenzene** | 3.72E-02 | 3.55E-02 | 3.99E-02 | 3.41E-02 | 3.99E-02 | 3.41E-02 | 3.67E-02 |
| **Methacrylonitrile** | 4.71E-03 | 3.45E-03 | 9.42E-03 | 1.29E-02 | 7.36E-03 | 6.13E-03 | 6.75E-03 | **1,3,5-trimethylbenzene** | 2.17E-02 | 2.92E-02 | 5.31E-02 | 3.90E-02 | 5.31E-02 | 2.17E-02 | 3.57E-02 |
| **Trichloroethene** | 1.27E-03 | 1.48E-03 | 2.49E-03 | 2.16E-03 | 1.77E-03 | 1.75E-03 | 1.76E-03 | **Propionitrile** | 4.77E-02 | 2.80E-02 | 2.51E-02 | 1.74E-02 | 4.77E-02 | 1.74E-02 | 2.96E-02 |
| **1,2-dichlorobenzene** | 9.71E-04 | 1.69E-03 | 1.71E-03 | 1.41E-03 | 1.71E-03 | 1.19E-03 | 1.45E-03 | **1,2,3-trimethylbenzene** | 1.80E-02 | 2.32E-02 | 4.14E-02 | 3.16E-02 | 4.14E-02 | 1.80E-02 | 2.85E-02 |
| **Hexachloro-1,3-butadiene** | 2.58E-02 | 2.50E-02 | 2.67E-02 | 2.48E-02 | 2.67E-02 | 2.48E-02 | 2.56E-02 | **Hexachloro-1,3-butadiene** | 2.58E-02 | 2.50E-02 | 2.67E-02 | 2.48E-02 | 2.67E-02 | 2.48E-02 | 2.56E-02 |
| **1,3-dichlrorobenzene** | 2.54E-02 | 2.42E-02 | 2.48E-02 | 2.42E-02 | 2.54E-02 | 2.42E-02 | 2.46E-02 | **1,3-dichlrorobenzene** | 2.54E-02 | 2.42E-02 | 2.48E-02 | 2.42E-02 | 2.54E-02 | 2.42E-02 | 2.46E-02 |
| **1,2-dichlorobenzene** | 2.48E-02 | 2.41E-02 | 2.51E-02 | 2.42E-02 | 2.51E-02 | 2.41E-02 | 2.46E-02 | **1,2-dichlorobenzene** | 2.48E-02 | 2.41E-02 | 2.51E-02 | 2.42E-02 | 2.51E-02 | 2.41E-02 | 2.46E-02 |
| **1,2,4-trichlorobenzene** | 2.45E-02 | 2.43E-02 | 2.46E-02 | 2.43E-02 | 2.46E-02 | 2.43E-02 | 2.44E-02 | **1,2,4-trichlorobenzene** | 2.45E-02 | 2.43E-02 | 2.46E-02 | 2.43E-02 | 2.46E-02 | 2.43E-02 | 2.44E-02 |
| **4-Ethyltoluene** | 2.08E-02 | 2.14E-02 | 3.20E-02 | 2.09E-02 | 3.20E-02 | 2.08E-02 | 2.38E-02 | **4-Ethyltoluene** | 2.08E-02 | 2.14E-02 | 3.20E-02 | 2.09E-02 | 3.20E-02 | 2.08E-02 | 2.38E-02 |
| **1,1,2-trichloroethane** | 2.21E-02 | 1.97E-02 | 2.00E-02 | 1.87E-02 | 2.21E-02 | 1.87E-02 | 2.01E-02 | **1,1,2-trichloroethane** | 2.21E-02 | 1.97E-02 | 2.00E-02 | 1.87E-02 | 2.21E-02 | 1.87E-02 | 2.01E-02 |
| **Methacrylonitrile** | 2.57E-02 | 9.46E-03 | 1.51E-02 | 1.29E-02 | 2.57E-02 | 9.46E-03 | 1.58E-02 | **Methacrylonitrile** | 2.57E-02 | 9.46E-03 | 1.51E-02 | 1.29E-02 | 2.57E-02 | 9.46E-03 | 1.58E-02 |
| **n-butylbenzene** | 5.92E-03 | 7.33E-03 | 1.30E-02 | 8.56E-03 | 1.30E-02 | 5.92E-03 | 8.70E-03 | **n-butylbenzene** | 5.92E-03 | 7.33E-03 | 1.30E-02 | 8.56E-03 | 1.30E-02 | 5.92E-03 | 8.70E-03 |


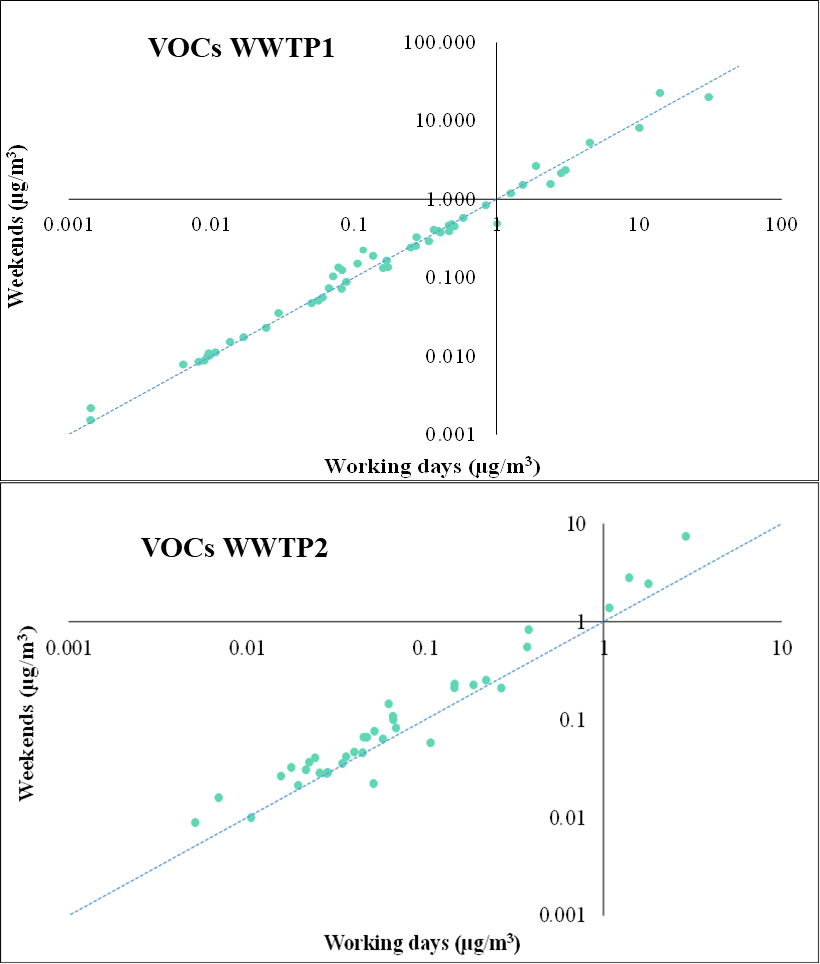


**Fig. S7*.*** Weekly average VOCs concentrations: weekend vs working days (Monday-Friday). Each point represents the experimental data of one of the detected compounds for the whole study.

**Table S4.** Summary of VOCs concentrations detected in both WWTPs with the highest and lowest average levels for each VOC, and the average for the whole study (in µg m^-3^). Ozone formation potential (OFP), secondary organic aerosol potential formation (SOAPF), carcinogenic risk (CR), non-carcinogenic risk (hazard index, HI) and odor impact values (OIV).

| **WWTP1** | **Max** | **Min** | **Average** | **OFP** | **SOAPF** | **CR** | **HI** | **OIV** | **WWTP2** | **Max** | **Min** | **Average** | **OFP** | **SOAPF** | **CR** | **HI** | **OIV** |
| --- | --- | --- | --- | --- | --- | --- | --- | --- | --- | --- | --- | --- | --- | --- | --- | --- | --- |
| **Benzoic acid** | 6.10E+01 | 6.38E+00 | 2.55E+01 |  |  |  |  |  | **Benzoic acid** | 2.66E+01 | 1.23E+01 | 1.78E+01 |  |  |  |  |  |
| **Toluene** | 3.38E+01 | 3.99E+00 | 1.89E+01 | 7.56E+01 | 1.02E+02 |  | 3.78E-03 | 1.53E+01 | **Toluene** | 4.91E+00 | 2.19E+00 | 3.13E+00 | 1.28E+01 | 1.73E+01 |  | 6.26E-04 | 1.53E+01 |
|  |  |  |  |  |  |  |  |  | **Decanal** | 8.88E+00 | 3.62E+00 | 6.51E+00 |  |  |  |  |  |
| **Methyl Acetate** | 1.42E+01 | 3.60E+00 | 6.75E+00 | 4.86E-01 |  |  |  |  |  |  |  |  |  |  |  |  |  |
| **Octanoic acid** | 4.90E+00 | 1.95E+00 | 2.76E+00 |  |  |  |  |  |  |  |  |  |  |  |  |  |  |
|  |  |  |  |  |  |  |  |  | **Nonanal** | 7.27E+00 | 3.30E+00 | 5.47E+00 |  |  |  |  |  |
|  |  |  |  |  |  |  |  |  | **Benzaldehyde** | 7.17E+00 | 3.85E+00 | 5.02E+00 |  |  |  |  |  |
| **Undecanal** | 1.74E-01 | 1.74E-01 | 1.74E-01 |  |  |  |  |  | **Undecanal** | 7.81E+00 | 2.39E+00 | 4.64E+00 |  |  |  |  |  |
| **2-Butanone** | 9.82E-01 | 5.51E-01 | 7.51E-01 |  |  |  |  |  | **2-Butanone** | 5.89E+00 | 1.69E+00 | 3.63E+00 |  |  |  |  |  |
| **Benzene** | 9.06E-01 | 7.48E-01 | 8.27E-01 | 5.95E-01 | 4.15E+00 | 2.76E-06 | 2.76E-02 |  | **Benzene** | 5.07E+00 | 1.65E+00 | 3.44E+00 | 2.22E+00 | 2.01E+00 | 1.15E-05 | 1.15E-01 |  |
| **Ethyl Acetate** | 2.97E+00 | 1.52E+00 | 1.98E+00 |  |  |  |  | 6.37E-01 | **Ethyl Acetate** | 6.52E+00 | 1.09E+00 | 3.18E+00 | 1.86E+00 |  |  |  | 6.37E-01 |
| **𝛂-pinene** | 3.69E+00 | 4.87E-01 | 2.28E+00 | 1.03E+01 | 2.14E+00 |  |  | 2.29E+01 | **𝛂-pinene** | 5.53E-01 | 1.96E-01 | 3.37E-01 | 1.45E+00 | 3.02E-01 |  |  | 2.29E+01 |
| **Acetophenone** | 2.09E+00 | 5.96E-01 | 1.22E+00 |  |  |  |  |  | **Acetophenone** | 1.56E+01 | 9.41E+00 | 1.15E+01 |  |  |  |  |  |
| **m-p Xylene** | 2.04E+00 | 1.03E+00 | 1.53E+00 | 1.20E+01 | 3.73E+00 |  | 2.12E-02 |  | **m-p Xylene** | 1.38E+00 | 4.20E-01 | 8.90E-01 | 1.44E+00 | **1.76E+00** |  | 6.15E-03 |  |
| **Butyl Acetate** | 6.78E+00 | 6.50E-01 | 2.70E+00 | 2.24E+00 |  |  |  |  |  |  |  |  |  |  |  |  |  |
|  |  |  |  |  |  |  |  |  | **Phenol** | 4.61E+00 | 1.89E+00 | 2.65E+00 |  |  |  |  |  |
| **Carbon tetrachloride** | 4.21E-01 | 3.67E-01 | 3.94E-01 | 0.00E+00 |  | 1.01E-06 | 3.94E-03 |  | **Carbon tetrachloride** | 2.19E+00 | 1.30E+00 | 1.57E+00 | 0.00E+00 |  | 4.03E-06 | 1.57E-02 |  |
| **o- Xylene** | 7.22E-01 | 4.52E-01 | 5.87E-01 | 4.48E+00 | 2.13E+00 |  |  | 3.58E-01 | **o-Xylene** | 5.14E-01 | 1.82E-01 | 3.39E-01 | 2.52E+00 | 1.70E+00 |  |  | 3.58E-01 |
| **Nonane** | 8.00E-01 | 3.01E-01 | 4.84E-01 | 3.77E-01 | 4.96E-02 |  |  | 4.22E-02 | **Nonane** | 6.89E-01 | 2.19E-01 | 4.62E-01 | 3.37E-01 | 4.44E-02 |  |  | 4.22E-02 |
|  |  |  |  |  |  |  |  |  | **Dodecanal** | 2.35E+00 | 7.17E-01 | 1.44E+00 |  |  |  |  |  |
|  |  |  |  |  |  |  |  |  | **Octanal** | 1.80E+00 | 7.26E-01 | 1.22E+00 |  |  |  |  |  |
|  |  |  |  |  |  |  |  |  | **Heptanal** | 1.40E+00 | 5.25E-01 | 9.24E-01 |  |  |  |  |  |
| **Decane** | 9.18E-01 | 2.54E-01 | 4.79E-01 | 3.26E-01 | 1.81E-01 |  |  | 1.34E-01 | **Decane** | 7.78E-01 | 1.72E-01 | 4.35E-01 | 2.72E-01 | 1.51E-01 |  |  | 1.34E-01 |
| **Ethylbenzene** | 5.93E-01 | 3.40E-01 | 4.67E-01 | 3.07E+00 | 2.81E+00 | 5.00E-07 | 1.79E-03 | 6.37E-01 | **Ethylbenzene** | 5.80E-01 | 1.69E-01 | 3.47E-01 | 2.19E+00 | 2.01E+00 | 3.72E-07 | 1.33E-03 | 6.37E-01 |
| **1-Butanol** | 8.17E-01 | 1.64E-01 | 4.37E-01 |  |  |  |  | 3.82E+00 | **1-Butanol** | 4.51E-01 | 2.02E-01 | 2.86E-01 |  |  |  |  | 3.82E+00 |
| **1,1,2-trichlorotrifluoroethane** | 7.55E-01 | 7.10E-01 | 7.32E-01 |  |  |  |  |  |  |  |  |  |  |  |  |  |  |
| **n-Hexane** | 5.99E-01 | 1.80E-01 | 3.90E-01 | 4.83E-01 | 2.10E-03 |  | 5.57E-04 |  |  |  |  |  |  |  |  |  |  |
| **Styrene** | 4.31E-01 | 2.18E-01 | 3.24E-01 | 1.26E+00 | 8.35E+00 |  | 7.28E-04 | 2.19E+00 | **Styrene** | 1.05E-01 | 7.26E-02 | 9.02E-02 | 1.67E-01 | 1.11E+00 |  | 9.02E-05 | 2.19E+00 |
| **Chloroform** | 1.62E-01 | 1.46E-01 | 1.54E-01 | 3.38E-03 |  |  | 1.57E-03 | 8.35E-03 | **Chloroform** | 1.73E+00 | 4.14E-01 | 8.49E-01 | 1.75E-02 |  |  | 8.67E-03 | 8.35E-03 |
|  |  |  |  |  |  |  |  |  | **Hexanal** | 8.26E-01 | 2.93E-01 | 5.54E-01 |  |  |  |  |  |
| **n-Heptane** | 3.09E-01 | 2.66E-01 | 2.88E-01 | 3.08E-01 |  |  |  |  | **n-Heptane** | 1.81E-01 | 6.95E-02 | 1.07E-01 | 1.23E-01 |  |  |  |  |
| **1,2,4-trimethylbenzene** | 3.59E-01 | 1.77E-01 | 2.68E-01 | 2.38E+00 | 2.98E-01 |  |  | 4.58E-01 | **1,2,4-trimethylbenzene** | 1.63E-01 | 7.10E-02 | 1.11E-01 | 1.04E+00 | 1.30E-01 |  |  | 4.58E-01 |
| **Undecane** | 4.59E-01 | 1.44E-01 | 2.46E-01 | 1.50E-01 | 2.15E-01 |  |  | 4.46E-02 | **Undecane** | 8.09E-01 | 1.47E-01 | 4.00E-01 | 2.25E-01 | 3.22E-01 |  |  | 4.46E-02 |
| **Dodecane** | 2.94E-01 | 1.06E-01 | 1.67E-01 | 9.21E-02 | 3.12E-01 |  |  | 2.20E-01 | **Dodecane** | 3.70E-01 | 1.63E-01 | 3.03E-01 | 1.63E-01 | 5.53E-01 |  |  | 2.20E-01 |
| **2-nitropropane** | 2.94E-01 | 2.83E-02 | 1.61E-01 | 1.77E-02 |  | 3.87E-07 | 8.06E-03 |  |  |  |  |  |  |  |  |  |  |
|  |  |  |  |  |  |  |  |  | **1,2-dichloroethane** | 3.24E-01 | 1.91E-01 | 2.54E-01 |  |  |  |  |  |
| **n-Octane** | 1.76E-01 | 1.19E-01 | 1.48E-01 | 1.64E-01 | 6.38E-03 |  |  | 1.72E-02 | **n-Octane** | 1.54E-01 | 9.52E-02 | 1.31E-01 | 1.46E-01 | 5.69E-03 |  |  | 1.72E-02 |
| **Camphene** | 2.01E-01 | 5.85E-02 | 1.28E-01 | 5.79E-01 |  |  |  |  | **Camphene** | 1.07E-01 | 3.02E-02 | 6.37E-02 | 2.79E-01 |  |  |  |  |
| **p-isopropyltoluene** | 1.62E-01 | 5.50E-02 | 1.08E-01 | 4.81E-01 |  |  |  |  | **p-isopropyltoluene** | 3.15E-01 | 4.74E-02 | 1.89E-01 | 8.91E-01 |  |  |  |  |
| **n-propylbenzene** | 1.37E-01 | 4.00E-02 | 8.87E-02 | 1.80E-01 | 5.25E-01 |  |  | 5.62E+00 | **n-propylbenzene** | 5.65E-02 | 2.56E-02 | 3.74E-02 | 7.73E-02 | 2.26E-01 |  |  | 5.62E+00 |
| **Tetrahydrofuran** | 1.03E-01 | 7.19E-02 | 8.75E-02 | 3.77E-01 |  |  |  |  | **Tetrahydrofuran** | 3.28E-01 | 7.39E-02 | 1.46E-01 | 5.42E-01 |  |  |  |  |
| **Tetrachloroethene** | 8.95E-02 | 7.59E-02 | 8.27E-02 |  |  | 9.22E-09 | 2.07E-03 | 1.60E-02 | **Tetrachloroethene** | 2.53E-01 | 1.33E-01 | 1.80E-01 |  |  | 2.01E-08 | 4.51E-03 | 1.60E-02 |
| **1,3,5-trimethylbenzene** | 1.08E-01 | 5.06E-02 | 7.93E-02 | 9.32E-01 | 5.78E-02 |  |  |  | **1,3,5-trimethylbenzene** | 5.31E-02 | 2.17E-02 | 3.57E-02 | 4.41E-01 | 2.74E-02 |  |  |  |
|  |  |  |  |  |  |  |  |  | **Pentanal** | 3.47E-01 | 1.17E-01 | 2.27E-01 |  |  |  |  |  |
| **Napthalene** | 9.23E-02 | 4.40E-02 | 6.82E-02 | 2.28E-01 |  | 9.93E-07 | 2.27E-02 |  | **Naphthalene** | 9.32E-02 | 6.34E-02 | 8.08E-02 | 2.53E-01 |  |  | 1.18E-06 | 2.69E-02 |
| **4-Ethyltoluene** | 8.99E-02 | 2.89E-02 | 5.94E-02 | 2.64E-01 | 2.24E-01 |  |  | 1.47E+00 | **4-Ethyltoluene** | 3.20E-02 | 2.08E-02 | 2.38E-02 | 1.04E-01 | 8.85E-02 |  |  | 1.47E+00 |
| **2-Ethyltoluene** | 7.72E-02 | 3.23E-02 | 5.47E-02 | 3.06E-01 | 2.80E-01 |  |  | 1.52E-01 | **2-Ethyltoluene** | 6.61E-02 | 3.52E-02 | 4.81E-02 | 2.69E-01 | 2.46E-01 |  |  | 1.52E-01 |
| **1,2,3-trimethylbenzene** | 6.89E-02 | 3.03E-02 | 4.96E-02 | 5.94E-01 | 3.62E-02 |  |  |  | **1,2,3-trimethylbenzene** | 4.14E-02 | 1.80E-02 | 2.85E-02 | 3.41E-01 | 2.08E-02 |  |  |  |
| **Isopropylbenzene** | 4.02E-02 | 2.39E-02 | 3.21E-02 | 8.08E-02 | 1.65E-01 |  |  | 7.82E-01 | **Isopropylbenzene** | 7.96E-02 | 5.25E-02 | 6.06E-02 | 1.54E-01 | 3.15E-01 |  |  | 7.82E-01 |
| **Bromoform** | 3.36E-02 | 1.40E-02 | 2.38E-02 |  |  | 1.12E-08 |  |  | **Bromoform** | 9.61E-02 | 7.37E-02 | 8.42E-02 |  |  | 3.97E-08 |  |  |
| **1,2-dichloropropane** | 1.94E-02 | 1.46E-02 | 1.70E-02 | 4.93E-03 |  |  |  |  | **1,2-dichloropropane** | 7.34E-02 | 5.53E-02 | 6.62E-02 | 1.96E-02 |  |  |  |  |
| **Bromodichloromethane** | 1.67E-02 | 1.14E-02 | 1.40E-02 |  |  |  |  |  | **Bromodichloromethane** | 5.71E-02 | 3.86E-02 | 4.57E-02 |  |  |  |  |  |
| **Propionitrile** | 1.16E-02 | 9.68E-03 | 1.07E-02 |  |  |  |  |  | **Propionitrile** | 4.77E-02 | 1.74E-02 | 2.96E-02 |  |  |  |  |  |
| **Chlorobenzene** | 1.44E-02 | 5.63E-03 | 1.00E-02 |  |  |  | 1.00E-05 |  | **Chlorobenzene** | 7.28E-02 | 2.09E-02 | 4.41E-02 |  |  |  | 4.41E-05 |  |
|  |  |  |  |  |  |  |  |  | **Dibromomethane** | 6.26E-02 | 3.72E-02 | 4.94E-02 |  |  |  |  |  |
| **n-butylbenzene** | 1.27E-02 | 6.84E-03 | 9.77E-03 | 2.31E-02 |  |  |  | 2.11E-01 | **n-butylbenzene** | 1.30E-02 | 5.92E-03 | 8.70E-03 | 2.17E-02 |  |  |  | 2.11E-01 |
| **Trichloroethene** | 1.77E-03 | 1.75E-03 | 1.76E-03 |  |  |  |  | 8.46E-05 | **1,1,1-trichloroethane** | 5.06E-02 | 3.57E-02 | 4.24E-02 |  |  |  |  |  |
| **Dibromochloromethane** | 1.16E-02 | 7.52E-03 | 9.53E-03 |  |  |  |  |  | **Trichloroethene** | 6.09E-02 | 2.29E-02 | 3.78E-02 |  |  |  |  | 8.46E-05 |
|  |  |  |  |  |  |  |  |  | **1,4-dichlorobenzene** | 3.99E-02 | 3.41E-02 | 3.67E-02 |  |  |  |  |  |
| **Methacrylonitrile** | 7.36E-03 | 6.13E-03 | 6.75E-03 |  |  |  |  | 8.26E-04 | **Methacrylonitrile** | 2.57E-02 | 9.46E-03 | 1.58E-02 |  |  |  |  | 8.26E-04 |
| **sec-butylbenzene** | 9.63E-03 | 7.84E-03 | 8.73E-03 |  |  |  |  |  |  |  |  |  |  |  |  |  |  |
| **Dibromomethane** | 9.26E-03 | 7.10E-03 | 8.18E-03 |  |  |  |  |  |  |  |  |  |  |  |  |  |  |
| **1,2-dichlorobenzene** | 1.71E-03 | 1.19E-03 | 1.45E-03 | 2.58E-04 |  |  |  |  | **1,2-dichlorobenzene** | 2.51E-02 | 2.41E-02 | 2.46E-02 | 4.39E-03 |  |  |  |  |
|  |  |  |  |  |  |  |  |  | **Hexachloro-1,3-butadiene** | 2.67E-02 | 2.48E-02 | 2.56E-02 |  |  |  |  |  |
|  |  |  |  |  |  |  |  |  | **1,3-dichlrorobenzene** | 2.54E-02 | 2.42E-02 | 2.46E-02 |  |  |  |  |  |
|  |  |  |  |  |  |  |  |  | **1,2,4-trichlorobenzene** | 2.46E-02 | 2.43E-02 | 2.44E-02 |  |  |  |  |  |
|  |  |  |  |  |  |  |  |  | **1,1,2-trichloroethane** | 2.21E-02 | 1.87E-02 | 2.01E-02 |  |  |  |  |  |
|  |  |  |  |  |  |  |  |  | **tert-butylbenzene** | 6.26E-03 | 3.24E-03 | 4.45E-03 |  |  |  |  |  |

**Table S5.** PM measures, carried out with portable equipment at the sample point and bioreactor in both WWTPs.

| **WWTP1** | **PM_1_ (µg/m^3^)** | **PM_2.5_ (µg/m^3^)** | **PM_10_ (µg/m^3^)** | **WWTP2** | **PM_1_ (µg/m^3^)** | **PM_2.5_ (µg/m^3^)** | **PM_10_ (µg/m^3^)** |
| --- | --- | --- | --- | --- | --- | --- | --- |
| **Sample point** | 6.6 | 5 | 12 | **Sample point** | 1.5 | 5 | 10 |
| **Bioreactor** | 9.2 | 20 | 53 | **Bioreactor** | 1.2 | 5 | 11 |
| **Sample point** | 1.9 | 11 | 17 | **Sample point** | 2.4 | 4 | 12 |
| **Bioreactor** | 10.9 | 21 | 60 | **Bioreactor** | 2.1 | 4 | 13 |
| **Sample point** | 1.6 | 5 | 11 | **Sample point** | 1.4 | 8 | 7 |
| **Bioreactor** | 13.9 | 24 | 65 | **Bioreactor** | 1.3 | 6 | 8 |
| **Sample point** | 6.6 | 5 | 12 | **Sample point** | 3.5 | 9 | 13 |
| **Bioreactor** | 14.9 | 20 | 53 | **Bioreactor** | 3.3 | 10 | 13 |


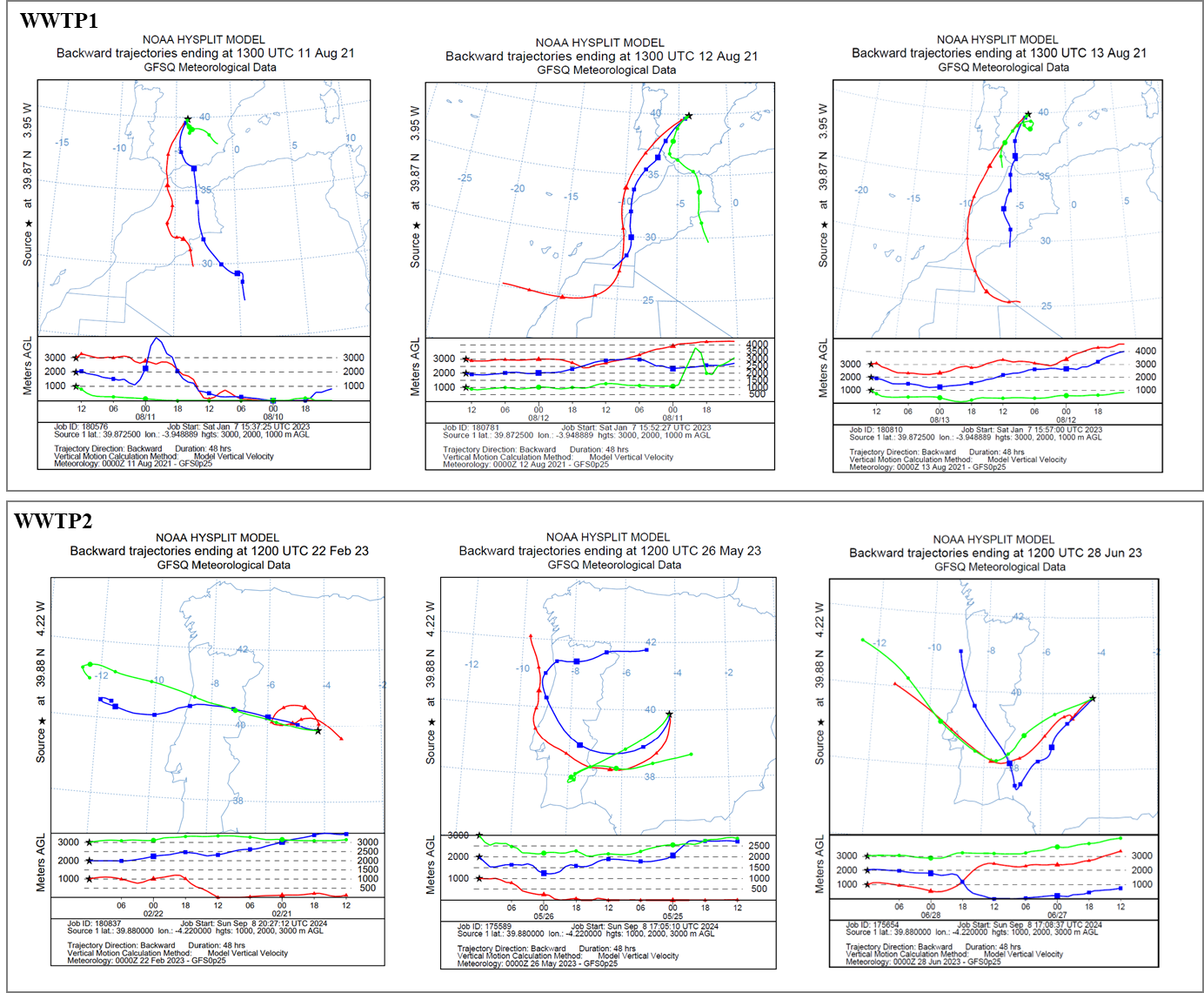


**Fig. S8**. 48 hours back trajectories of air masses for the WWTPs obtained for altitudes 1000, 2000 and 3000 meters.

**Fig. S9.** Weekly average PM concentrations: Sundays vs working days (Monday-Saturday).

**Table S6**. Average*,* minimum, and maximum concentrations of metals associated with PM_2.5_ identified at WWTPs.

| **Element** | **WWTP1** | | | | **WWTP2** | | | | **Recomended/Limit values** | |
| --- | --- | --- | --- | --- | --- | --- | --- | --- | --- | --- |
|  | **Min (ng/m^3^)** | **Max**  **(ng/m^3^)** | **Average (ng/m^3^)** | **SD** | **Min (ng/m^3^)** | **Max (ng/m^3^)** | **Average (ng/m^3^)** | **SD** | **WHO ^a^** | **UE ^b^** |
| **Na** | 4.75E+02 | 1.16E+04 | 5.71E+04 | 4.67E+03 | 5.81E+02 | 8.51E+03 | 3.11E+03 | 4.43E+03 |  |  |
| **Zn** | ND | 5.66E+03 | 2.48E+03 | 2.15E+03 | ND | 4.67E+03 | 1.69E+03 | 3.43E+03 |  |  |
| **K** | ND | 4.66E+03 | 2.15E+03 | 1.90E+03 | ND | 3.32E+03 | 1.58E+03 | 1.91E+03 |  |  |
| **Al** | 2.06E+02 | 4.21E+03 | 1.77E+03 | 1.40E+03 | ND | 1.43E+03 | 3.67E+02 | 9.29E+02 |  |  |
| **Ca** | 1.63E+02 | 8.43E+02 | 5.24E+02 | 2.84E+02 | 1.55E+01 | 8.36E+02 | 1.75E+02 | 3.31E+02 |  |  |
| **Mg** | 9.60E+00 | 4.49E+02 | 1.10E+02 | 1.67E+02 | 4.26E+01 | 1.29E+02 | 8.43E+01 | 4.20E+01 |  |  |
| **Fe** | 1.85E+01 | 8.23E+01 | 3.95E+01 | 2.49E+01 | 2.21E+01 | 7.62E+02 | 3.61E+02 | 3.72E+02 |  |  |
| **Cu** | 3.00E-01 | 4.30E+00 | 1.60E+00 | 1.40E+00 | 1.28E+00 | 6.50E+01 | 1.89E+01 | 2.68E+01 |  |  |
| **Mn** | 4.00E-01 | 2.30E+00 | 1.20E+00 | 7.00E-01 | ND | 1.99E+01 | 6.08E+00 | 1.04E+01 | 1.50E+02 |  |
| **Ni** | ND | 4.20E+00 | 1.20E+00 | 1.90E+00 | ND | 1.30E+01 | 1.82E+00 | 5.75E+00 | 2.50E+01 | 2.00E+01 |
| **Pb** | ND | 2.90E+00 | 1.10E+00 | 1.20E+00 | ND | 7.94E+00 | 2.14E+00 | 4.37E+00 | 5.00E+02 | 5.00E+02 |
| **Cr** | 1.00E-01 | 1.30E+00 | 6.00E-01 | 4.00E-01 | 2.25E-01 | 2.53E+00 | 1.13E+00 | 1.46E+00 |  |  |
| **As** | 2.00E-01 | 9.00E-01 | 4.00E-01 | 2.00E-01 | ND | 2.10E+00 | 2.26E-01 | 9.23E-01 | 6.60E+00 | 6.00E+00 |
| **Se** | ND | 6.00E-01 | 3.00E-01 | 2.00E-01 | ND | 1.03E+00 | 5.09E-01 | 4.78E-01 |  |  |
| **Co** | ND | 2.00E-01 | 1.00E-01 | 1.00E-01 | ND | 5.27E-01 | 1.19E-01 | 2.48E-01 |  |  |
| **Cd** | ND | ND | ND | ND | ND | 4.10E-01 | 9.61E-02 | 1.93E-01 | 5.00E+00 | 5.00E+00 |
| **Hg** | ND | ND | ND | ND | ND | ND | ND | ND | 1.00E+03 |  |
| **Total elements (ng/m^3^)** | 2.33E+03 | 2.45E+04 | 1.28E+04 | 9.36E+03 | 1.39E+03 | 1.61E+04 | 7.62E+03 | 9.91E+03 |  |  |

Note. ND: not detectable; SD: standard deviation; Min: minimum; Max: maximum; **a** Values obtained from WHO (2000) for metals; **b** Values obtained from UE (2021) for metals.

**Fig. S10.** Back trajectories calculated with HYSPLIT and SplitR Library Software during the samplings. Colors indicate the relative amount or frequency of trajectories at each cell.

**Fig. S11.** The calculated values of Ei (blue bars) and EF (orange squares) for metals in PM_2.5_. Blue line (Ei = 40) indicates the limit between low and moderate ecological risk. Orange line (EF = 10) indicates the limit between crustal and anthropogenic origin.

**Table S7**. Environmental and health risk indicators related to TEs.

| **WWTP2**  **WWTP1** | | | | | |
| --- | --- | --- | --- | --- | --- |
| **Element** | **Cancer Risk (CR)** | **Non-Carcinogenic (HI)** | **Cancer Risk (CR)** | **Non-Carcinogenic (HI)** | **IARC Group** |
| **As** | 7.73E-07 | 2.80E-02 | 3.37E-07 | 1.22E-02 | 1 |
| **Se** | 0.00E+00 | 1.33E-05 | 0.00E+00 | 2.45E-05 | 3 |
| **Na** | 0.00E+00 | 0.00E+00 | 0.00E+00 | 0.00E+00 |  |
| **Mg** | 0.00E+00 | 0.00E+00 | 0.00E+00 | 0.00E+00 |  |
| **Al** | 0.00E+00 | 0.00E+00 | 0.00E+00 | 0.00E+00 |  |
| **K** | 0.00E+00 | 0.00E+00 | 0.00E+00 | 0.00E+00 |  |
| **Ca** | 0.00E+00 | 0.00E+00 | 0.00E+00 | 0.00E+00 |  |
| **Mn** | 0.00E+00 | 4.03E-03 | 0.00E+00 | 1.30E-02 |  |
| **Fe** | 0.00E+00 | 0.00E+00 | 0.00E+00 | 0.00E+00 |  |
| **Co** | 1.46E-07 | 1.21E-03 | 1.25E-07 | 1.04E-03 | 2A |
| **Ni** | 1.21E-07 | 1.31E-02 | 1.85E-07 | 1.99E-02 | 2B |
| **Cu** | 0.00E+00 | 1.16E-05 | 0.00E+00 | 1.64E-04 |  |
| **Zn** | 0.00E+00 | 2.37E-03 | 0.00E+00 | 1.30E-03 |  |
| **Cd** | 0.00E+00 | 0.00E+00 | 5.18E-08 | 6.71E-03 | 1 |
| **Hg** | 0.00E+00 | 0.00E+00 | 0.00E+00 | 0.00E+00 | 3 |
| **Pb** | 3.70E-08 | 7.19E-03 | 6.79E-08 | 1.32E-02 | 2B |
